# Forkhead box K2 modulates epirubicin and paclitaxel sensitivity through FOXO3a in breast cancer

**DOI:** 10.1038/oncsis.2015.26

**Published:** 2015-09-07

**Authors:** G Nestal de Moraes, P Khongkow, C Gong, S Yao, A R Gomes, Z Ji, N Kandola, D Delbue, E P S Man, U S Khoo, A D Sharrocks, E W-F Lam

**Affiliations:** 1 Department of Surgery and Cancer, Imperial College London, Imperial Centre for Translational and Experimental Medicine (ICTEM), London, UK; 2 Laboratório de Hemato-Oncologia Celular e Molecular, Programa de Hemato-Oncologia Molecular, Instituto Nacional de Câncer (INCA), Rio de Janeiro, Brazil; 3 Department of Pathology, Li Ka Shing Faculty of Medicine, The University of Hong Kong, Hong Kong, China; 4 Faculty of Life Sciences, University of Manchester, Manchester, UK

## Abstract

The forkhead transcription factor FOXK2 has recently been implicated in cancer cell proliferation and survival, but a role in cancer chemotherapeutic drug resistance has hitherto not been explored. Here we demonstrate that FOXK2 has a central role in mediating the cytotoxic drug response in breast cancer. Clonogenic and cell viability assays showed that enhanced FOXK2 expression sensitizes MCF-7 breast cancer cells to paclitaxel or epirubicin treatment, whereas FOXK2 depletion by small interfering RNAs (siRNAs) confers drug resistance. Our data also showed that the activation of the tumour suppressor FOXO3a by paclitaxel and epirubicin is mediated through the induction of FOXK2, as depletion of FOXK2 by siRNA limits the induction of FOXO3a by these drugs in MCF-7 cells. Chromatin immunoprecipitation (ChIP) analysis showed that in response to drug treatment, FOXK2 accumulates and binds to the proximal *FOXO3a* promoter region in MCF-7 cells. Furthermore, we also uncovered that FOXK2 is deregulated and, therefore, can express at high levels in the nucleus of both the paclitaxel and epirubicin drug-resistant MCF-7 cells. Our results showed that ectopically overexpressed FOXK2 accumulates in the nuclei of drug-resistant MCF-7 cells but failed to be recruited to target genes, including FOXO3a. Crucially, we found that FOXO3a is required for the anti-proliferative and epirubicin-induced cytotoxic function of FOXK2 in MCF-7 cells by sulphorhodamine and clonogenic assays. The physiological importance of the regulation of FOXO3a by FOXK2 is further confirmed by the significant correlations between FOXO3a and FOXK2 expression in breast carcinoma patient samples. Further survival analysis also reveals that high nuclear FOXK2 expression significantly associates with poorer clinical outcome, particularly in patients who have received conventional chemotherapy, consistent with our finding that FOXK2 is deregulated in drug-resistant cells. In summary, our results suggest that paclitaxel and epirubicin target the FOXK2 to modulate their cytotoxicity and deregulated FOXK2 confers drug resistance.

## Introduction

Forkhead box K (FOXK) proteins are a subgroup of the Forkhead transcription factors, characterized by a conserved DNA-binding domain^1^ known to regulate a diverse range of biological processes, such as differentiation, survival, metabolism, senescence, apoptosis and cell cycle progression.^2^ FOXK2 is one of the two FOXK isoforms in mammals and was first identified as a NFAT-like interleukin-binding factor.^3^ Compared with the related FOXK1, little is known about the biological function and mechanism of regulation of FOXK2. It has been demonstrated that FOXK2 can be phosphorylated by cyclin/CDK complexes in a cell cycle-dependent manner.^4^ FOXK2 has also been shown to associate with AP-1 transcription factor to modify chromatin, thus enabling AP-1 binding to its target genes.^5, 6^ In that study, genome-wide Chromatin immunoprecipitation (ChIP)-seq analysis shows that FOXK2 can regulate a wide range of gene networks, particularly those involved in cell adhesion and motility, metabolism and, interestingly, apoptosis and cancer.^5, 6^ Recently, it has been reported that FOXK2 can interact with the polycomb complex molecules and recruit the BAP-1 tumour suppressor protein to the chromatin,^6, 7^ further confirming that FOXK2 might function in modifying the chromatin structure.

Paclitaxel and epirubicin belong to the taxane and anthracycline classes of chemotherapeutic agents, respectively. Although these drugs are highly effective and commonly used for the management of breast cancer, chemoresistance commonly arises and accounts for treatment failure. Deregulation of expression and activity of some of the tumour-suppressive FOX transcription factors, such as FOXO3a, has been linked extensively to breast cancer initiation and progression, as well as drug resistance.^8, 9^ FOXO3a has a crucial role in mediating the cytotoxic effects of chemotherapeutic agents in breast cancer through the modulation of downstream transcriptional targets.^10^ It has been demonstrated that FOXO3a expression and nuclear translocation are induced in response to paclitaxel^11, 12^ and doxorubicin treatment,^13^ suggesting that enhancing FOXO3a activity might potentiate the sensitivity of breast cancer cells to chemotherapy.^10^ However, there is hitherto no description of the role of FOXK2 in breast cancer drug resistance and that it is not known whether FOXK2 is modulated following treatment with chemotherapeutic agents. Considering these observations, we speculated that FOXK2 could have a role in drug resistance in breast cancer. We show here that FOXK2 regulates FOXO3a to modulate drug sensitivity and that deregulation of expression and activity of FOXK2 confers paclitaxel and epirubicin resistance and associates with a poor clinical outcome in breast carcinoma patients.

## Results

### FOXK2 is differentially expressed in drug-sensitive and -resistant breast cancer cell lines

**Figure 1:**
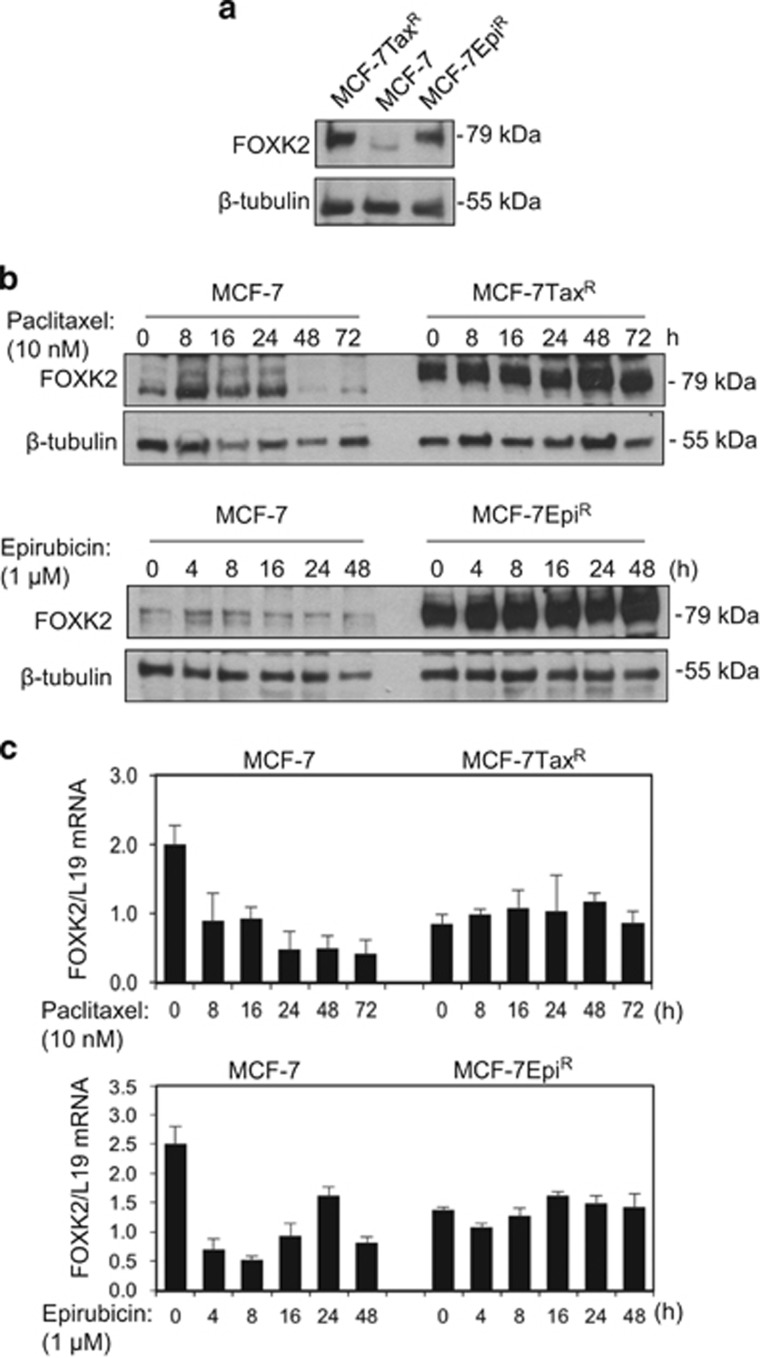
FOXK2 expression in sensitive and drug-resistant breast cancer cell lines. (**a**) MCF-7, MCF-7Tax^R^ and MCF-7Epi^R^ were harvested and their lysates subjected to western blot analysis, where FOXK2 and β-tubulin levels were determined. (**b**) MCF-7 and MCF-7Tax^R^ were treated with 10 nm paclitaxel for 0, 8, 16, 24, 48 and 72 h (upper panel). MCF-7 and MCF-7Epi^R^ were treated with 1 μm epirubicin for 0, 4, 8, 16, 24 and 48 h (lower panel). Cells were collected and subjected to western blot analysis. FOXK2 and β-tubulin levels were determined. (**c**) MCF-7 and MCF-7Tax^R^ were exposed to 10 nm paclitaxel for 0, 8, 16, 24, 48 and 72 h (upper panel). MCF-7 and MCF-7EpiR were exposed to 1 μm epirubicin for 0, 4, 8, 16, 24 and 48 h (lower panel). The levels of FOXK2 mRNA transcript were determined by qRT–PCR after normalization against L19. Bars represent average±s.d..

Recent evidence suggests that FOXK2 may have a role in cancer development.^4, 14^ However, the regulation and expression of FOXK2 in breast cancer and its role in drug resistance have hitherto not been explored. To address this, we first compared the expression patterns of FOXK2 in MCF-7 cells and its drug-resistant counterparts MCF-7Tax^R^ and MCF-7Epi^R^ cell lines. Western blotting analysis showed that FOXK2 is expressed consistently at higher levels in the paclitaxel- and epirubicin-resistant cells compared with the parental MCF-7 cells (Figure 1a). We next examined its expression in the wild-type and resistant MCF-7 cells in response to paclitaxel and epirubicin treatment, respectively. Although FOXK2 expression was low in MCF-7 cells, it was induced in response to paclitaxel and epirubicin treatment and then declined at later time points (Figure 1b; Supplementary Figure S1), most likely due to global protein degradation as a result of cell death.^15, 16, 17, 18, 19^ By contrast, FOXK2 expression was constitutively high and remained unchanged in the resistant cell lines following paclitaxel and epirubicin treatment (Figure 1b). Conversely, quantitative real-time PCR (qRT–PCR) analysis revealed that FOXK2 mRNA levels were consistently low in the resistant cell lines compared with sensitive MCF-7 cells (Figure 1c). Corroborating the protein data, FOXK2 mRNA levels were modulated upon paclitaxel and epirubicin treatment of MCF-7, but remained stable in the resistant cell lines (Figure 1c). Together, these results suggest that there are distinct differences in FOXK2 expression patterns in drug-sensitive and -resistant cell lines, with FOXK2 protein inducible by both paclitaxel and epirubicin in the drug-sensitive MCF-7 cells but not the resistant cells. The data also show that FOXK2 expression in drug-sensitive and -resistant MCF-7 cell lines is not primarily regulated at the transcriptional levels. We also obtained results to suggest that FOXK2 is predominantly located in the nucleus of breast cancer cell lines, regardless of their drug resistance and treatment (Supplementary Figures S2 and S3).

### FOXK2 depletion promotes resistance to paclitaxel and epirubicin in breast cancer cells

**Figure 2:**
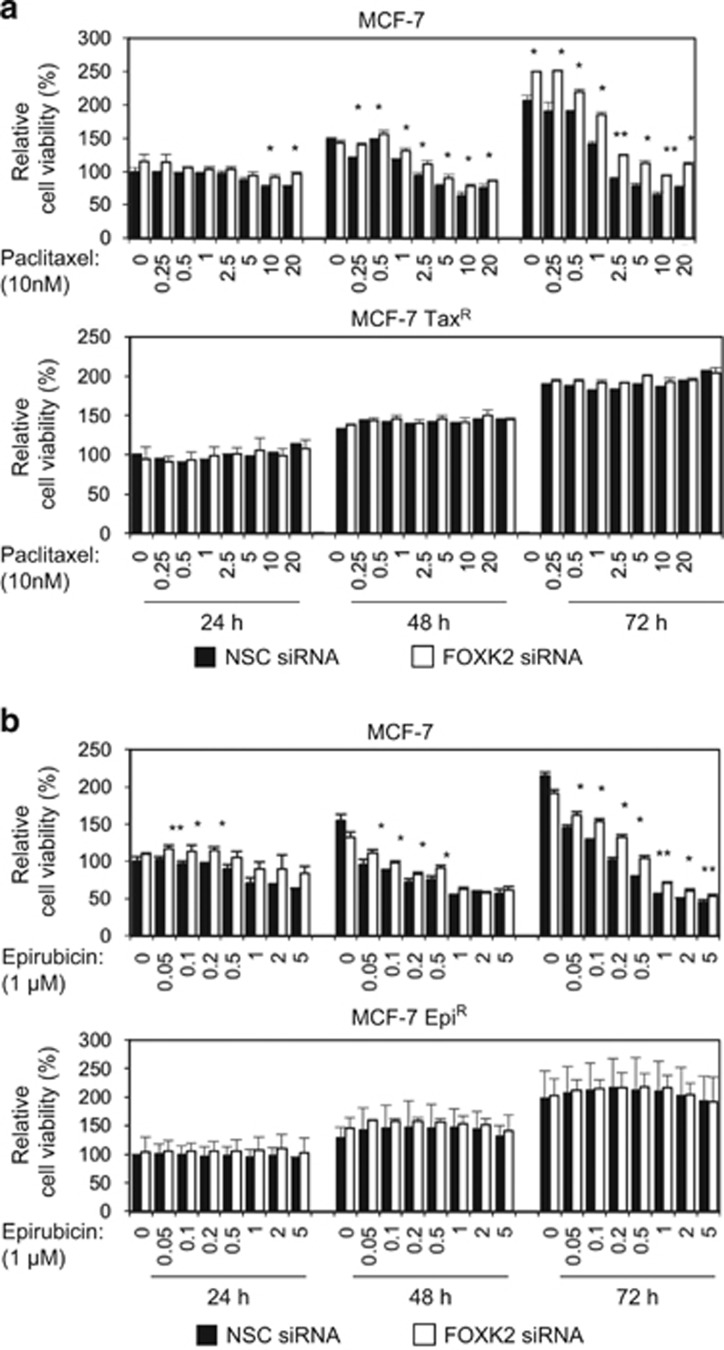
FOXK2 depletion promotes resistance to paclitaxel and epirubicin in sensitive but not resistant breast cancer cells. (**a**) MCF-7 and MCF-7Tax^R^ transfected with NSC (non-silencing control) and FOXK2 siRNA independently were seeded in 96-well plates and treated with increasing concentrations of paclitaxel for 24, 48 and 72 h. Viability was measured by SRB assay. (**b**) MCF-7 and MCF-7Epi^R^ transfected with NSC and FOXK2 siRNA were seeded in 96-well plates and treated with increasing concentrations of epirubicin for 24, 48 and 72 h. Viability was measured by SRB assay and compared between NSC and FOXK2 siRNA samples. The graphs are representative of three experiments. Statistical significance was determined by Student's *t*-test (two-sided; *P⩽0.05, **P⩽0.01, significant).

**Figure 3:**
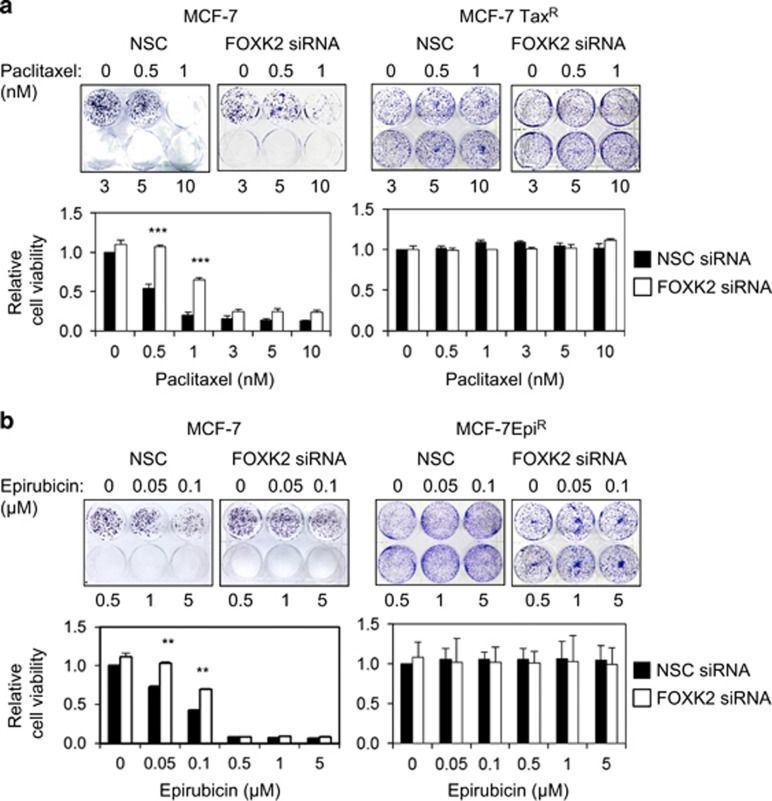
FOXK2 depletion promotes resistance to paclitaxel and epirubicin in sensitive but not resistant breast cancer cells. (**a**) MCF-7 and MCF-7Tax^R^ cells and (**b**) MCF-7 and MCF-7Epi^R^ cells were transfected with NSC (non-silencing control) and FOXK2 siRNA, seeded in six-well plates and treated with increasing concentrations of paclitaxel. After 48 h of incubation with the drugs, cells were cultured in fresh media, grown for around 14 days and stained with crystal violet. The graphs are representative of three experiments. Statistical significance was determined by Student's *t*-test (two-sided; **P⩽0.01, ***P⩽0.001, significant).

To investigate the role of FOXK2 in breast cancer drug resistance, FOXK2 expression was depleted using small interfering RNA (siRNA) in both sensitive and resistant cell lines, which were then treated with a range of concentrations of the chemotherapeutic drugs and assayed for their drug response through short- and long-term cell viability assays. Sulphorhodamine (SRB) assays showed that FOXK2-silenced MCF-7 cells were more resistant to both paclitaxel (Figure 2a, upper panel) and epirubicin (Figure 2b, upper panel). In the MCF-7Tax^R^ and MCF-7Epi^R^ resistant cell lines, we observed no effect of FOXK2 knockdown on drug response (Figures 2a and b, lower panels). Corroborating these data, there was an increase in the colony-formation capacity of FOXK2-depleted MCF-7 cells, particularly at 0.5 and 1 nm of paclitaxel and 0.05 and 0.1 μm of epirubicin treatment (Figures 3a and b, left panels). In agreement, we could not observe any changes in the colony-formation capacity of resistant cell lines in response to drug treatment (Figures 3a and b, right panels).

### FOXK2 regulates FOXO3a expression in drug-sensitive breast cancer cells

**Figure 4:**
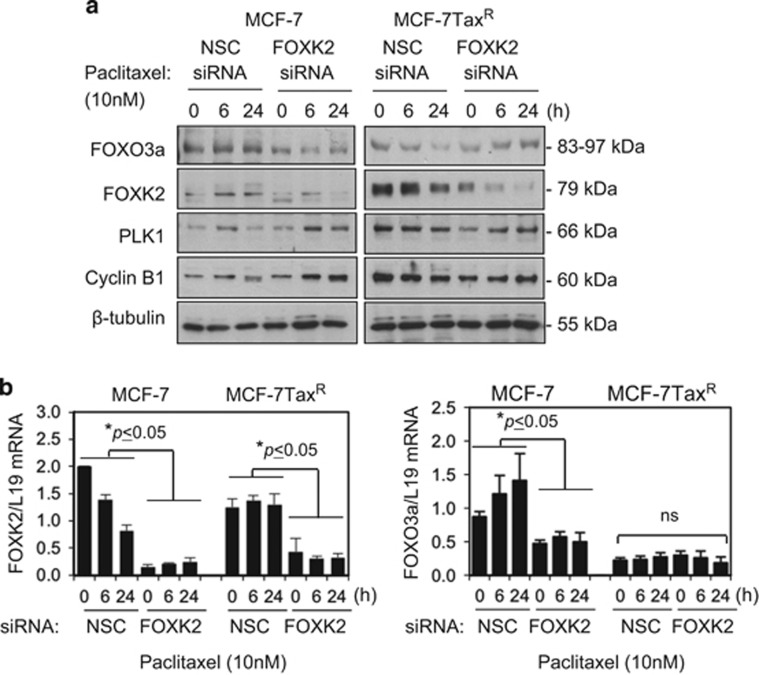
Depletion of FOXK2 transcription factor results in FOXO3a downregulation. (**a**) MCF-7 and MCF-7Tax^R^ were harvested and subjected to western blot analysis, where FOXK2, FOXO3a, PLK1, cyclin B1 and β-tubulin levels were determined. (**b**) MCF-7 and MCF-7Tax^R^ were transfected with NSC (non-silencing control) and FOXK2 siRNA and exposed to 10 nm paclitaxel for 0, 6 and 24 h. The levels of FOXK2 and FOXO3a mRNA transcripts were determined by qRT–PCR after normalization against L19. Bars represent average±s.d. Statistical significance was determined by Student's *t*-test (two-sided; *P⩽0.05, ⩽ significant). NS, not significant.

Previous studies demonstrated that FOXK2 cross-talks with the tumour suppressor FOXO3a to regulate cell proliferation and survival.^20^ Given that FOXO3a has been shown to mediate the cytostatic and cytotoxic functions of taxanes and anthracyclines,^11, 12, 15, 21^ together these findings led us to hypothesize that FOXK2 might target *FOXO3a* expression to induce breast cancer cell proliferation arrest and cell death in response to these conventional anticancer chemotherapeutics. To test this conjecture, we investigated FOXO3a expression following FOXK2 knockdown. The results showed that FOXK2 depletion resulted in a reduction of FOXO3a expression in the paclitaxel-sensitive MCF-7 but not in the resistant MCF-7Tax^R^ cells (Figure 4a; Supplementary Figure S4). We also analysed the expression of cyclin B1 and Polo-like kinase 1 (PLK1), targets known to be repressed by FOXO3a.^22^ Consistently, cyclin B1 and PLK1 were found to be upregulated in FOXK2-depleted MCF-7 cells, especially at 24 h after paclitaxel treatment (Figure 4a). We also found that FOXK2 knockdown resulted in downregulation in FOXO3a mRNA levels in MCF-7 cells, indicative of the transcriptional regulation of FOXO3a by FOXK2 (Figure 4b). By comparison, we did not observe substantial changes in FOXO3a and its downstream targets in the resistant MCF-7Tax^R^ cells upon FOXK2 depletion, suggesting FOXK2 is deregulated in the MCF-7Tax^R^ cells (Figures 4a and b). Together these results suggest that FOXK2 knockdown promotes paclitaxel and epirubicin resistance, which might involve the modulation of FOXO3a expression.

### Overexpression of FOXK2 enhances FOXO3a expression to sensitize breast cancer cells to paclitaxel

**Figure 5:**
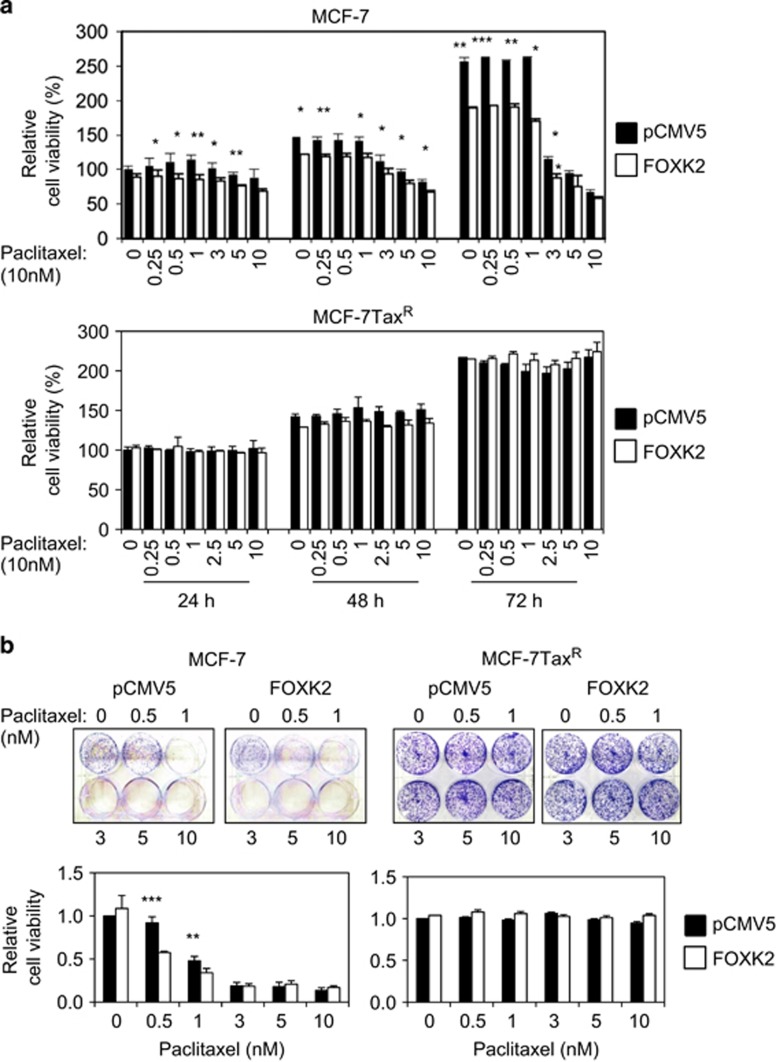
Overexpression of wild-type FOXK2 sensitizes MCF-7 cells to paclitaxel treatment. (**a**) MCF-7 and MCF-7Tax^R^ cells were transfected with the empty vector (pCMV5) and the wild-type FOXK2 vector, seeded in 96-well plates and treated with increasing concentrations of paclitaxel. After 24, 48 and 72 h of incubation with the drugs, cell viability was assessed by SRB assay. (**b**) MCF-7 and MCF-7Tax^R^ were transfected with the empty vector (pCMV5) and the wild-type FOXK2 vector, seeded in six-well plates and treated with increasing concentrations of paclitaxel. After 48 h of incubation with the drugs, cells were cultured in fresh media, grown for around 14 days and stained with crystal violet. The graphs are representative of three experiments. Statistical significance was determined by Student's *t*-test (two-sided; *P⩽0.05, **P⩽0.01, ***P⩽0.001, significant).

**Figure 6:**
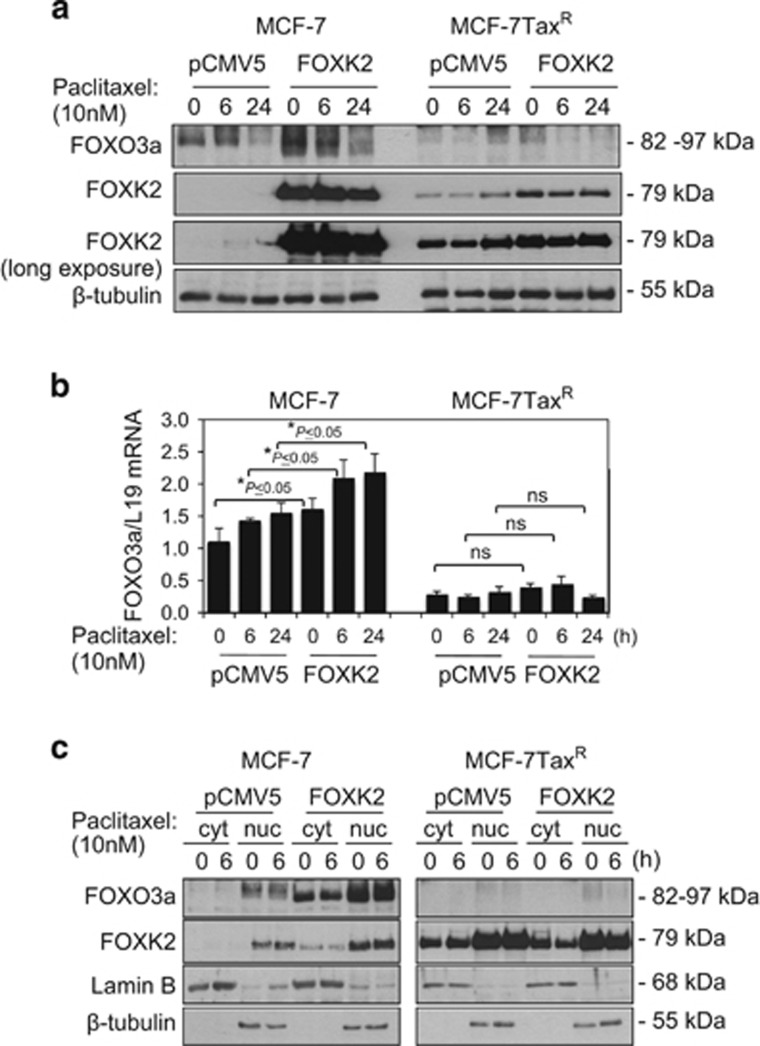
FOXK2 overexpression upregulates FOXO3a expression. (**a**) MCF-7 and MCF-7Tax^R^ cells were collected following FOXK2 overexpression and subjected to western blot analysis, where FOXK2, FOXO3a and β-tubulin levels were determined. (**b**) MCF-7 and MCF-7Tax^R^ cells were transfected with the empty vector (pCMV5) and the wild-type FOXK2 vector sequences and exposed to 10 nm paclitaxel for 0, 6 and 24 h. The levels of FOXO3a and FOXM1 mRNA transcript were determined by qRT–PCR after normalization against L19. Bars represent average±s.d. from three independent experiments. Statistical significance was determined by Student's *t*-test (*P⩽0.05, significant). (**c**) MCF-7 and MCF-7Tax^R^ were treated with 10 nm paclitaxel for 0 and 6 h and harvested for subcellular fractionation. Afterwards, cytoplasmic (cyt) and nuclear (nuc) fractions were subjected to western blot analysis, and FOXK2, β-tubulin (cytoplasmic loading control) and Lamin B (nuclear loading control) levels were determined. NS, not significant.

We next overexpressed FOXK2 and examined its effects on paclitaxel resistance. Consistent with FOXK2 knockdown results, our short-term viability data show that FOXK2-overexpressing MCF-7 cells displayed a more sensitive phenotype towards paclitaxel than cells transfected with the empty vector (Figure 5a, upper panel). To further confirm this result, we performed clonogenic assay and observed that, at 0.5 and 1 nm of paclitaxel, FOXK2 overexpression could also inhibit the colony-formation capacity of MCF-7 cells in response to paclitaxel (Figure 5b, left panel). Interestingly, induction of FOXK2 overexpression in MCF-7Tax^R^ resistant cells could not sensitize them to paclitaxel treatment, as examined by both short- and long-term cell viability assays (Figure 6a, lower panel and Figure 6b, right panel). To investigate whether the lack of effect of FOXK2 overexpression on sensitizing MCF-7Tax^R^ cells to paclitaxel was a result of mislocalization of wild-type FOXK2, we analysed FOXK2 localization in sensitive and resistant cells and found that FOXK2 was localized predominantly in the nucleus of both MCF-7 and MCF-7Tax^R^ cells (Supplementary Figure S5). This result indicates that the differences in effects of FOXK2 overexpression in sensitive and resistant breast cancer cell lines are not owing to deregulated FOXK2 subcellular localization. We next studied the effects of FOXK2 overexpression on FOXO3a protein and mRNA expression levels, as well as its subcellular localization. Our results showed that FOXK2 overexpression caused an induction of FOXO3a protein (Figure 6a) and mRNA (Figure 6b) levels in MCF-7, but not in MCF-7Tax^R^ cells, further confirming that FOXK2 regulates FOXO3a expression in paclitaxel-sensitive cells. Consistently, the levels of FOXO3a transcription factor were increased in the nucleus of FOXK2-overexpressing MCF-7 cells (Figure 6c). By contrast, FOXO3a nuclear expression levels remained low in the MCF-7Tax^R^ resistant cells, where we observed no effect of FOXK2 overexpression on drug response. To further confirm the role of FOXK2 in paclitaxel sensitivity and response, we overexpressed FOXK2 in the moderately paclitaxel-resistant MDA-MB-231 breast cancer cells (Supplementary Figure S6a) and found the FOXK2-overexpressing MDA-MB-231 cells were more sensitive to paclitaxel treatment compared with control MDA-MB-231 cells (Supplementary Figure S6b) and had decreased colony-formation capacities in response to paclitaxel (Supplementary Figure S6c). Consistently, these effects were associated with upregulation of FOXO3a at the protein and mRNA levels (Supplementary Figures S6d and S6e). Conversely, knockdown of FOXK2 in MDA-MB-231 cells by using siRNA also resulted in the cells becoming more resistant to paclitaxel (Supplementary Figure S7). Collectively, these findings suggest that FOXK2 targets FOXO3a expression in breast cancer cells to enhance sensitivity to paclitaxel.

### FOXK2 regulates FOXO3a expression through a forkhead response element in its promoter

**Figure 7:**
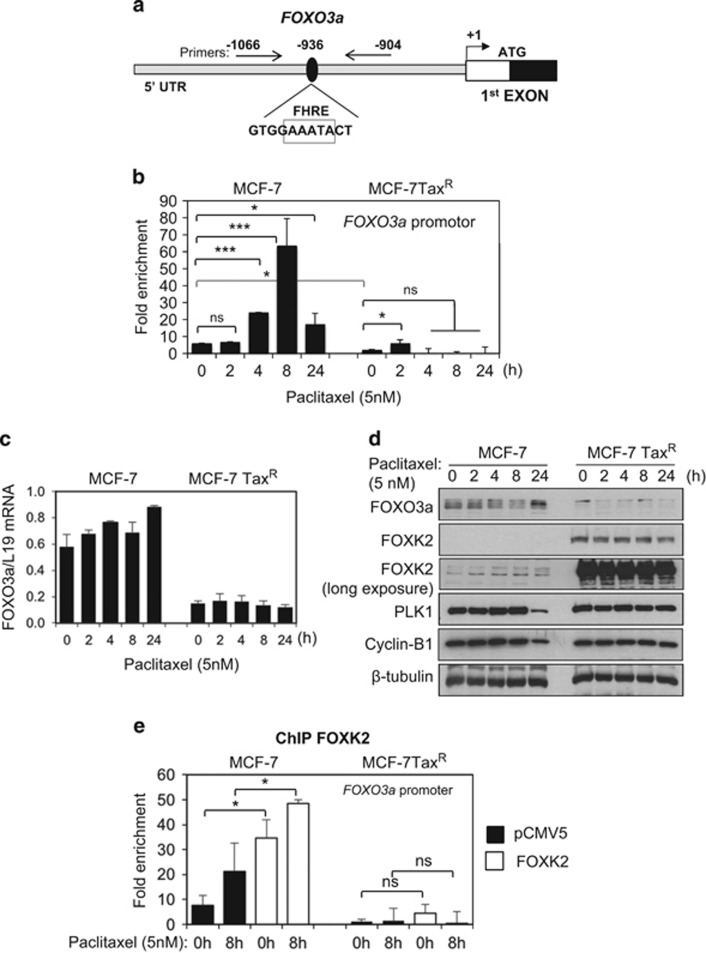
FOXK2 binds to the *FOXO3a* promoter in MCF-7 cells. (**a**) Chromatin immunoprecipitation (ChIP) analysis of FOXO3a promoter. A pair of primers amplifying the forkhead responsive elements (FHRE)-binding-site-containing *FOXO3a* promoter region (-1066/-907 bp) was designed. (**b**) MCF-7 and MCF-7Tax^R^ treated with 5 nm paclitaxel for 0, 2, 4, 8 and 24 h were used for chromatin immunoprecipitation assays using the IgG as negative control and anti-FOXK2 antibody. After reversal of cross-linking, the co-immunoprecipitated DNA was amplified by qRT–PCR, using primers amplifying the FOXK2 binding-site-containing region. (**c**) MCF-7 and MCF-7Tax^R^ cells were exposed to 5 nm paclitaxel for 0, 2, 4, 8 and 24 h and the levels of FOXO3a mRNA transcript were determined by qRT–PCR after normalization against L19. Bars represent average±s.d. from two independent experiments. (**d**) After treatment of MCF-7 and MCF-7Tax^R^ cells with 5 nm paclitaxel for 0, 2, 4, 8 and 24 h, the cells were lysed for western blot analysis, where FOXK2, FOXO3a, PLK1, cyclin B1 and β-tubulin levels were determined. (**e**) MCF-7 and MCF-7Tax^R^ cells transfected with the empty vector (pCMV5) and wild-type FOXK2 were used for chromatin immunoprecipitation assays using the IgG as negative control and anti-FOXK2 antibody. After reversal of cross-linking, the co-immunoprecipitated DNA was amplified by qRT–PCR, using primers amplifying the FOXK2 binding-site-containing region in FOXO3a promoter. Statistical significance was determined by Student's t-test (*P⩽0.05, ****P⩽0.005; ns., non-significant).

Analysis of a recently published ChIP-sequencing data revealed that in U2OS cells, FOXK2 is recruited to the promoter region of the human *FOXO3a* gene, containing a forkhead response element (FHRE;^5^ Figure 7a). We therefore next investigated whether FOXK2 regulates FOXO3a expression directly through binding to the *FOXO3a* promoter. For this purpose, we studied the occupancy of the FHRE region of the *FOXO3a* promoter by FOXK2 using ChIP in MCF-7 cells in response to paclitaxel treatment. Our results showed that the binding of FOXK2 to the FHRE region of the *FOXO3a* promoter increased in response to paclitaxel treatment in MCF-7 cells (Figure 7b). Corroborating our previous data, only low levels of FOXK2 binding were detected on the endogenous *FOXO3a* promoter in MCF-7Tax^R^ cells either at basal levels or following paclitaxel treatment (Figure 7b), further confirming that FOXK2 is unable to drive FOXO3a expression in paclitaxel-resistant cells. In agreement, treatment with 5 nm of paclitaxel provoked a substantial induction of FOXO3a expression at the protein and mRNA levels in MCF-7, but not MCF-7Tax^R^ cells (Figures 7c and d). We also analysed the expression of PLK1 and cyclin B1 as downstream targets of FOXO3a and found that their expression was downregulated particularly at 24 h post-paclitaxel treatment, when FOXO3a expression increased. To further confirm our findings on the regulation of *FOXO3a* expression by FOXK2, we overexpressed FOXK2 in both MCF-7 and MCF-7Tax^R^ cell lines and analysed its recruitment to the *FOXO3a* promoter FHRE by RT–qPCR following ChIP. Our RT–qPCR results showed that overexpression of FOXK2 caused an increase in FOXK2 binding to the *FOXO3a* promoter in MCF-7 cells (Figure 7e). In addition, the binding of FOXK2 to *FOXO3a* promoter was further enhanced by paclitaxel treatment, confirming that FOXK2 transcriptional activity is inducible by drug treatment. In contrast, there were no significant increases in the recruitment of FOXK2 to the *FOXO3a* promoter in MCF-7Tax^R^ cells following FOXK2 overexpression, suggesting that FOXK2 is deregulated and inactive in the resistant cells. Together, these data show that FOXK2 can directly bind to the *FOXO3a* promoter and regulate its expression in the paclitaxel-sensitive, but not the resistant, breast cancer cells.

### FOXK2 restricts cell proliferation and enhances drug sensitivity through targeting FOXO3a

**Figure 8:**
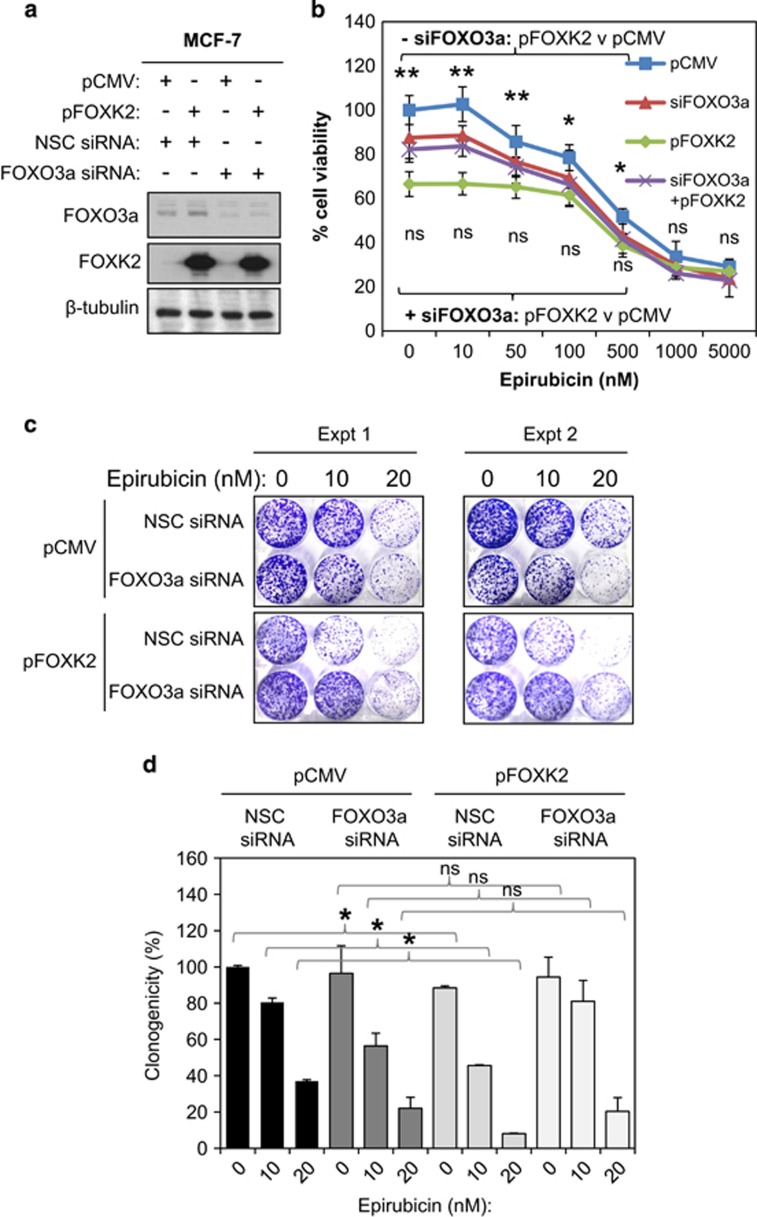
FOXO3a is required for the anti-proliferative and epirubicin-induced cytotoxic function of FOXK2. MCF-7 cells were transiently transfected with either the control pCMV or FOXK2 and non-silencing control (NSC) siRNA or the FOXO3a siRNA smart pool. (**a**) Twenty-four hours after transfection, protein lysates were prepared from these cells and then analysed for the expression of FOXK2, FOXO3a and β-tubulin (**b**) The transfected cells were also treated with a range of doses of epirubicin (0–5000 nm) and their proliferative rates assayed by SRB assay at 48 and 72 h after epirubicin treatment. Statistical significance was determined by Student's *t*-test (two-sided; *P⩽0.05, **P⩽0.01, NS, not significant) by comparing the proliferation rates of cells transfected with pFOXK2 with the control pCMV transfected cells. (**c**) Twenty-four hours after transfection, the 2000 cells were seeded in six-well plates, treated with 0, 10 or 20 nm of epirubicn, grown for 15 days and then stained with crystal violet. Two representative independent experiments are shown (top panel). (**d**) The result (bottom panel) represents the average of three independent experiments±s.d. Statistical significance was determined by Student's *t*-test (two-sided; *P⩽0.05, **P⩽0.01, NS, not significant).

FOXO3a has been shown to mediate the cytotoxic function of anthracyclines, including epirubicin, and, therefore, likely has a central role in mediating the anti-proliferative function of FOXK2. To test this idea, we overexpressed FOXK2 in MCF-7 cells with or without FOXO3a depletion (Figure 8a) and tested their effects on cell proliferation upon epirubicin treatment. More specifically, the proliferation rate and clonogenicity of these transfected MCF-7 cells in response to a range of epirubicin concentrations were monitored by SRB and clonogenic assays, respectively. The cell proliferation assays showed that overexpression of FOXK2 or FOXO3a depletion significantly limited MCF-7 cell proliferation compared with the control MCF-7 cells, transfected with non-silencing siRNA pool and the empty expression vector (Figure 8b; Supplementary Figure S8a). This is somewhat unexpected as FOXO3a is a well-established tumour suppressor;^2, 9^ however, it has also been shown to have a role in cell renewal and expansion.^23, 24^ Despite the fact that both FOXK2 overexpression and FOXO3a depletion independently repressed cell proliferation, MCF-7 cells with simultaneous FOXK2 overexpression and FOXO3a depletion displayed similar proliferative rates as the cells with FOXO3a silenced only, suggesting that FOXO3a is essential for the anti-proliferative function of FOXK2. The result showed that overexpression of FOXK2 significantly increases the sensitivity of MCF-7 cells to epirubicin (Figure 8a and Supplementary Figure S8a). Furthermore, we also found that FOXK2 overexpression enhanced the sensitivity of MCF-7 cells to epirubicin but had no appreciable anti-proliferative effects on MCF-7 cells with FOXO3a depleted in terms of drug sensitivity (Figure 8b and Supplementary Figure S8b). Consistently, clonogenic assays also revealed that although FOXK2 ectopic expression could potentiate the cytotoxic function of epirubicin in the control MCF-7 cells with FOXO3a expression, FOXK2 overexpression had no significant effects in MCF-7 cells with FOXO3a depleted (Figures 8c and d). Together these results indicate that FOXO3a is a key target of FOXK2 and that FOXO3a mediates the anti-proliferative effects and cytotoxic function of FOXK2 in response to epirubicin.

### FOXK2 nuclear expression correlates with FOXO3a expression and clinical outcome in breast cancer patients

**Figure 9:**
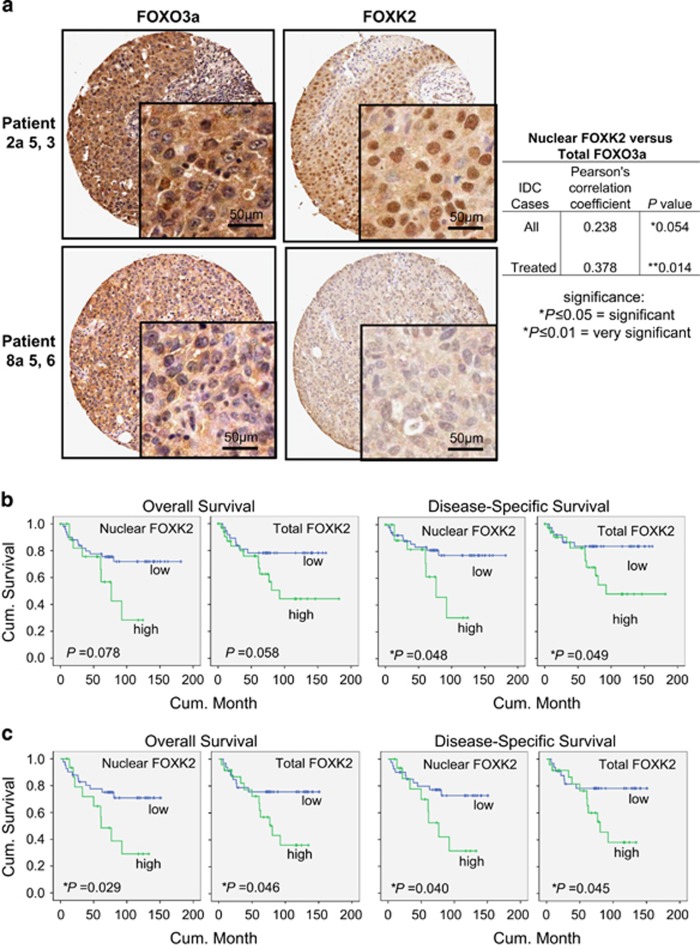
Positive correlation between FOXK2 and FOXO3a expression in breast cancer patients. (**a**) FOXK2 and FOXO3a expression was assessed by immunohistochemistry using tissue-microarray constructed from 116 breast cancer patient samples. Representative staining images of one patient with high FOXK2 and FOXO3a expression and one with low expression are shown. Magnification: Images ( × 20) and Insets ( × 100). FOXK2 was expressed predominantly in the nucleus, while FOXO3a staining were detected in both nuclear and cytoplasmic compartments. Positive correlation between nuclear FOXK2 and total FOXO3a expression was observed. Statistical analysis revealed that nuclear FOXK2 was marginally significantly correlated with total FOXO3a expression (Pearson coefficient *r*=0.238, *P=0.054, chi-square test) in all IDC cases and very significantly correlated with total FOXO3a expression (Pearson coefficient *r*=0.378, **P=0.014; chi-square test) in IDC patients that received chemotherapy (**b**) Kaplan–Meier survival analysis (SPSS) of all IDC patients showed that nuclear FOXK2 overexpression associated with poorer survival (*n*=109). (**c**) Kaplan–Meier survival analysis of IDC patients received chemotherapy showed that nuclear FOXK2 overexpression significantly associated with poorer survival (*n*=65). Significance: *P⩽0.05, significant; **P⩽0.01, very significant. IDC, invasive ductal carcinoma.

To ascertain further the physiological and clinical importance of the regulation of FOXO3a by FOXK2 in breast cancer, FOXO3a and FOXK2 expression was assessed by immunohistochemistry in 86 invasive ductal carcinoma patient samples, of which 46 received anthracyclin and taxane chemotherapy treatment (Figure 9a; Supplementary Figure S10). Consistent with our finding from the breast cancer cell lines that FOXK2 expresses primarily in the nucleus, all the patients had predominantly nuclear FOXK2 expression. Statistical analysis of the immunohistochemical results revealed that nuclear, but not cytoplasmic or total, FOXK2 expression is marginally correlated with total FOXO3a expression (Pearson coefficient *r*=0.238, *P=0.054; *P⩽0.05=significant) in all the invasive ductal carcinoma cases (Figure 9a; Supplementary Figure S9). However, nuclear FOXK2 was strongly and significantly associated with total FOXO3a (Pearson coefficient *r*=0.378, **P=0.014; **P⩽0.01, very significant) in patients that received chemotherapy (Figure 9a; Supplementary Figure S8). This corroborates the finding from the breast cancer cell lines and further suggests that FOXK2 directly regulates FOXO3a transcription. Moreover, nuclear FOXK2 expression was also significantly correlated with oestrogen receptor status and tumour stage (Supplementary Figure S11). Further survival analysis showed that nuclear and total FOXK2 expression was associated with overall survival but not statistically significant (Kaplan–Meier analysis; log-rank test, *P*=0.078 and *P*=0.058, respectively; Figure 9b; Supplementary Figure S12). However, of all invasive ductal carcinoma patients, nuclear and total FOXK2 expression was significantly correlated with disease-specific survival (log-rank test, *P=0.048 and *P=0.049, respectively; Figure 9b; Supplementary Figure S12). Of patients that received chemotherapy, elevated nuclear and total FOXK2 was significantly associated with poorer outcome (log-rank test, *P=0.029 and *P=0.048 for overall survival, and *P=0.040 and *P=0.045 for disease-specific survival, respectively; Figure 9c; Supplementary Figure S12). The significance of FOXK2 in survival analyses provides further evidence for its involvement in breast cancer progression and drug response. These results are in accordance with our *in vitro* observations of overexpression of FOXK2 in drug-resistant cell lines and further suggest that constitutively high expression levels of nuclear FOXK2 is associated with drug resistance and poor clinical outcome.

## Discussion

The development of drug resistance is a major obstacle to successful cancer chemotherapy and is associated with limited therapeutic options and poorer prognosis in cancer patients. Recent evidence has linked FOXK2 to cancer cell proliferation and survival,^25^ but hitherto, a role in cancer chemotherapeutic drug resistance has not been established. In this study, we examined the role and regulation of FOXK2 in mediating the cell proliferation and chemotherapeutic drug response in breast cancer. We identified FOXK2 as a key mediator of the cytotoxic function of paclitaxel and epirubicin as well as a modulator of drug sensitivity in breast cancer. In the presence of paclitaxel or epirubicin treatment, clonogenic and cell viability assays showed that enhanced expression of FOXK2 inhibits cell proliferation and clonogenic growth of the drug-sensitive MCF-7 breast carcinoma cells. Conversely, FOXK2 depletion by siRNA increased cell viability and clonogenicity, and conferred resistance to these conventional cancer chemotherapeutic agents in MCF-7 cells. Notably, although FOXK2 depletion or overexpression causes profound changes in cell proliferation and viability in MCF-7 cells upon paclitaxel and epirubicin treatment, it has little effect in untreated MCF-7 cells. This suggests that the ability of FOXK2 to modulate paclitaxel and epirubicin sensitivity is not merely a reflection of the anti-proliferative function of FOXK2.

Our results also reveal that FOXK2 controls FOXO3a expression in response to paclitaxel and epirubicin. Our data show that FOXO3a activation by paclitaxel and epirubicin is mediated through the induction of FOXK2, as depletion of FOXK2 by siRNA impairs the induction of FOXO3a by paclitaxel and epirubicin in MCF-7 cells. Consistent with previous observations,^4, 14^ we show that FOXK2 is predominantly expressed in the nucleus, regardless of their drug resistance and treatment. Upon treatment with these cytotoxic agents, we found that FOXK2 remains in the nucleus, and this is associated with the induction of FOXO3a transcription and expression in MCF-7 and MDA-MB-231 cells. We also show by ChIP experiments that the *FOXO3a* promoter is a direct target of FOXK2 and binding is accompanied by or associated with FOXO3a expression and proliferative arrest. Together these results suggest that conventional chemotherapeutic agents, including anthracyclines and taxanes, induces FOXK2 to accumulate in the nucleus, where it induces the expression of downstream targets, including FOXO3a, a crucial mediator of the cytotoxic and cytostatic actions of anticancer chemotherapeutic agents in breast cancer cells^13, 15^ (Supplementary Figure S13). Furthermore, we also uncovered that FOXK2 is deregulated in both the paclitaxel and epirubicin drug-resistant MCF-7 cells and consequently can accumulate at high levels in these cells. Accordingly, cytoplasmic/nuclear protein fractionation, confocal microscopy and ChIP experiments revealed that ectopically overexpressed wild-type FOXK2 protein accumulates in the nucleus of the drug-resistant MCF-7Tax^R^ and MCF-7Epi^R^ cells but fails to be recruited to the target gene FOXO3a. This inability of FOXK2 to be recruited to its target genes, such as FOXO3a, is likely to occur at the post-translation levels. Alternatively, this can also be that the target genes are not accessible to FOXK2 as a result of epigenetic changes in the resistant cells. Indeed, post-translational modifications have previously been shown to modulate the transcriptional activity of FOXK2. For example, FOXK2 has been shown to be phosphorylated by cyclin-dependent kinases (CDKs) and hyperphosphorylated FOXK2 is less stable.^4^ However, the CDK-mediated phosphorylation is unlikely to be responsible for the inactivation of FOXK2 in the drug-resistant cells as FOXK2 is overexpressed but inactive in the drug-resistant cells.

The physiological relevance of the regulation of FOXO3a by FOXK2 is further underscored by the significant correlations between FOXO3a and FOXK2 expression in invasive ductal breast carcinoma patient samples. Intriguingly, survival analysis reveals that nuclear FOXK2 expression significantly associates with poorer clinical outcome, particularly in patients that have received conventional chemotherapy. This observation is in line with our findings in the paclitaxel- and epirubicin-resistant MCF-7 cells that in the drug-resistant breast cancer cells, the high levels of nuclear FOXK2 are deregulated and inactive, which will ultimately impact on chemotherapeutic drug response and patient survival. Collectively, our results suggest that FOXK2 mediates the cytotoxic drug response and functions as a tumour suppressor, which is often deregulated in drug-resistant cancer cells. However, FOXK2 has also been proposed to be required for cell proliferation.^14^ The reason for this apparent discrepancy is unclear but it could be that the cell models used in this study are mostly untransformed cells. Conversely, in concordance with our findings, ectopic FOXK2 expression has been shown to induce apoptosis in the osteosarcoma U2OS cells.^4^ Moreover, FOXK2 has also been shown to recruit BAP-1, an important tumour suppressor, to the chromatin and to promote local deubiquitination during DNA damage response.^6^

FOXM1 is a forkhead box transcription factor transcriptionally repressed by FOXO3a, which has been demonstrated to be a key modulator of the anticancer therapeutics, including taxanes and anthracylines.^10, 26, 27, 28, 29^ Accordingly, FOXM1 has been shown to confer genotoxic agent resistance through promoting the expression of targets, including *NBS1* (*Nebrin*), *XRCC1*, *BRCA2*, *CHEK1* (*CHK1*), *RAD51* and *BRIP1*,^15, 17, 30, 31^ important for DNA damage signalling and repair, to limit the genotoxic effects. In addition, FOXM1 can also promote resistance to taxanes and other mitotic modulators through driving the expression of the microtubulin-associated kinesins, such as KIF20A, at the transcriptional level.^19^ In consequence, FOXO3a can impact the cytotoxic function of FOXK2 through down-modulating FOXM1 transcription and activity.^10^ Thus, FOXK2 deregulation can lead to FOXO3a downregulation and FOXM1 overexpresssion. In agreement, FOXM1 has been demonstrated to be expressed at constitutively high levels in the MCF-7Tax^R^ and MCF-7Epi^R^ cells where FOXK2 is deregulated.^16, 17, 18, 19^

In summary, our findings suggest that paclitaxel and epirubicin target the FOXK2-FOXO3a axis to modulate their cytotoxic function and deregulated FOXK2 may confer drug resistance. We also present compelling evidence to show that drug-resistant breast cancer cells are able to tolerate high levels of nuclear FOXK2 because FOXK2 is deregulated and fails to be recruited to targets genes, such as FOXO3a. These data provide novel insights into the underlying mechanisms of chemotherapy resistance and have implications for the development of predictive biomarkers and novel chemotherapeutic strategies for drug resistance.

## Materials and methods

### Cell culture

The human breast cancer cell lines MCF-7 and MDA-MB-231 originated from the American Type Culture Collection (Manassas, VA, USA) and were acquired from the Cell Culture Service from Cancer Research UK, where they were authenticated. The cell lines were cultured in Dulbecco's modified Eagle's medium supplemented with 10% fetal calf serum, 100 U/ml penicillin/streptomycin and 2 mm glutamine and maintained in a humidified incubator with 10% CO_2_ at 37 °C. The MCF-7Tax^R^ and MCF-7Epi^R^ resistant cell lines were originated from MCF-7 and maintained in 50 nm paclitaxel and 17 μm doxorubicin, respectively.^16^

### Plasmids

The plasmids encoding human FOXK2 pCMV5-FOXK2WT and the empty vector pCMV5 have previously been described.^4^ Expression plasmid transfections were performed with FuGENE 6 (Roche, IN, USA), according to manufacturers' instructions. The cells were collected and seeded for drug treatment following 24 h of transfection.

### Western blot and antibodies

Western blotting was done in whole-cell extracts prepared by lysing cells in lysis buffer as previously described.^21^ The antibodies recognizing FOXK2 (A301-729A) and total FOXO3a (07-702) were purchased from Bethyl Labs (Cambridge Bioscience Ltd, Cambridge, UK) and Millipore (Hertfordshire, UK), respectively. Cyclin B1 (sc-752), PLK1 (sc-17783), Lamin B (sc-6217) and β-tubulin (sc-9104) antibodies were obtained from Santa Cruz Biotechnology (Santa Cruz; Insight Biotechnology, Middlesex, UK). Primary antibodies were detected using horseradish peroxidase-linked anti-mouse, anti-rabbit or anti-goat conjugates (Dako, Glostrup, Denmark) and visualized using the ECL detection system (PerkinElmer, Coventry, UK).

### Quantitative real-time PCR

The extraction of total RNA was performed with the RNeasy Mini Kit (Qiagen, Hilden, Germnay) and complementary DNA was synthetized by the Superscript III Method (Invitrogen, Paisley, UK), according to manufacturers's instructions. Transcript levels were analysed by qRT–PCR using the standard curve and normalized to ribosomal protein L19 mRNA levels, as previously described.^32^ The forward and reverse primers used were: L19-F: 5′-GCGGAAGGGTACAGCCAAT-3′ and L19-R: 5′-GCAGCCGGCGCAAA-3′, FOXK2-F: 5′-AGTCTCCAGTGAAGGCCGTA-3′ and FOXK2-R: 5′-CCCACCTTGTACCCTGAAGA-3′ and FOXO3a-F: 5′-TCTACGAGTGGATGGTGCGTT-3′ and FOXO3a-R: 5′- CGACTATGCAGTGACAGGTTGTG-3′.

### Gene silencing with siRNAs

For FOXK2 knockdown, cells were transfected with siRNA SMART pool reagents (Thermo Scientific Dharmacon; Life Technologies, Paisley, UK) using Oligofectamine (Invitrogen) based on manufacturer's recommendations. SMARTPool siRNAs used here were as follows: siRNA FOXK2 (L-008354-00-0010) and the non-silencing control siRNA (D-001810-10-50).

### ChIP assay

Following paclitaxel treatment and FOXK2 overexpression, cells were collected for the chromatin immunoprecipitation (ChIP) assay as previous described.^19^ The reaction was run in 7900 HT Fast Real-time PCR System (Applied Biosystems, Life Technologies Ltd, Paisley, UK) and the cycling programme was 95 °C for 10 min followed by 40 cycles of 95 °C for 15 s, 60 °C for 30 s and 95 °C for 30 s, followed by a dissociation step. The pair of primers used for ChIP was: FOXO3a-F, 5′-ACCAACATCTTCGCCGTTC-3′ and FOXO3a-R, 5′-GGTGTCCGGTTCCCTGTTAG-3′, Each sample was assayed in triplicates, and the results were normalized to the IgG antibody.

### SRB assay

For the assessment of short-term changes in cell viability in drug-treated cells following FOXK2 knockdown and overexpression, we performed the SRB assay. Briefly, 3000 cells were seeded in 96-well plates and left to adhere for 24 h, after which paclitaxel or epirubicin was added and cells were maintained in culture for an additional 24, 28 and 72 h. Cells were then fixed with 100 μl of 40% trichloroacetic acid for 1 h at 4 °C, washed three times with distilled water and stained with 100 μl SRB solution (0.4% SRB diluted in 1% acetic acid) for 1 h. Afterwards, the plates were washed three times with 1% acetic acid and air-dried. Protein-bound dye was solubilized in 100 μl of 10 mm Tris solution and optical density was measured in a microplate reader at 492 nm (Sunrise, Tecan, Dorset, UK).

### Clonogenic assay

For clonogenic assay, a total of 2000 MCF-7 and MCF-7Epi^R^ cells or 5000 MCF-7 and MCF-7Tax^R^ cells were seeded into six-well plates and left overnight for adherence, after which they were treated with increasing concentrations of paclitaxel and epirubicin as described.^19, 33^ After 48 h of incubation with the drugs, cells were cultured in fresh drug-free media and grown for ~14 days when colonies were formed. The colonies were washed three times with phosphate-buffered saline and fixed with 4% formaldehyde for 15 min at room temperature. After three additional washes with phosphate-buffered saline, the colonies were stained with 0.5% crystal violeta (Sigma-Aldrich UK, Poole, UK) for 1 h, washed with flowing water and air-dried. Then, 1 ml of 33% acetic acid was added to each well for crystal violet solubilization and optical density was measured at 592 nm using a microplate reader (Sunrise, Tecan, Dorset, UK).

### Tissue microarray, immunohistochemistry and staining scoring

These reagents and analyses have previously been described.^19, 33^ For details, see Supplementary Materials and Methods.

### Statistical analysis

Statistical analyses of data were conducted using the SPSS Statistics software (SPSS for Windows, version 17.0, SPSS Inc., IBM, London, UK). The Student's *t*-test was used to compare the differences between means from the two groups. The values of *P<0.05, **P<0.01 and ***P<0.005 were considered statistically significant. The Kaplan–Meier method was used to assess survival, which was compared between groups using the log-rank test. The association between FOXK2 and FOXO3a expression and clinical-pathological parameters was analysed by the chi-square test. A value of *P*<0.05 was considered statistically significant.
