# Supplementary material for: Forkhead box K2 modulates epirubicin and paclitaxel sensitivity through FOXO3a in breast cancer
Source: Oncogenesis. 2015 Sep 7;4(9):e167–. doi: 10.1038/oncsis.2015.26 (PMC4767938; doi:10.1038/oncsis.2015.26)
Supplement: Supplementary Information [file oncsis201526x1.docx]

**Supplementary Figure Legends**

**Supplementary Figure S1. Quantification of FOXK2 and FOXO3a expression in MCF-7, MCF-7Epi^R^  and MCF-7Tax^R^  cells in response to drug treatment.** FOXK2 and FOXO3a images from Figure 1b were quantified using ImageJ® analysis and plotted against signals for β-Tubulin.

**Supplementary Figure S2. FOXK2 is predominantly localised in the nucleus of both sensitive and resistant breast cancer cells. (a)** MCF-7 and MCF-7Tax^R^  cells were cultured on chamber slides and treated with 10 nM paclitaxel for 0, 6 and 24 h. Cells were then fixed and immunostained for FOXK2 (green). Immunostaining for α-tubulin (red) was performed to delineate cytoplasm and nuclei were counterstained with 4’-6-diamidino-2-phenyllindole (DAPI; blue). Images were acquired with Leica TCS SPS (x63 magnification). Images are representative of three independent experiments. **(b)** MCF-7 and MCF-7 Tax^R^ were treated with 10 nM paclitaxel for 0, 6 and 24 h and harvested for subcellular fractionation. Afterwards, cytoplasmic and nuclear fractions were subjected to western blot and FOXK2, β-tubulin (cytoplasmic loading control) and Lamin B (nuclear loading control) levels were determined.

**Supplementary Figure S3. FOXK2 nuclear localisation is not unique to MCF-7 cells and also is not modulated by epirubicin.** **(a)** MCF-7, MCF-7Tax^R^ and MCF-7Epi^R^ were harvested for subcellular fractioning and subjected to western blot analysis (left panel). MCF-7 and MCF-7Epi^R^ were treated with 1 µM epirubicin for 0, 6 and 24 h and harvested for subcellular fractionating. Cytoplasmic and nuclear fractions were subjected to western blot and FOXK2, β-tubulin (cytoplasmic loading control) and Lamin B (nuclear loading control) levels were determined (right panel). **(b)** MDA-MB-231 cells were cultured on chamber slides and treated with 10 nM paclitaxel for 0, 6 and 24 h. Cells were then fixed and immunostained for FOXK2 (green). Immunostaining for α-tubulin (red) was performed to delineate cytoplasm and nuclei were counterstained with 4’-6-diamidino-2-phenyllindole (DAPI; blue). Images were acquired with Leica TCS SPS (x63 magnification). Images are representative of three independent experiments. **(c)** MDA-MB-231 cells were treated with 10 nM paclitaxel for 0, 6 and 24 h and harvested for subcellular fractioting. Afterwards, cytoplasmic and nuclear fractions were subjected to western blot and FOXK2, β-tubulin (cytoplasmic) and Lamin B (nuclear) levels were determined.

**Supplementary Figure S4. Quantification of FOXK2, FOXO3a, Cyclin B1 and PLK1 expression in MCF-7 and MCF-7Tax^R^ cells with and without FOXK2 depletion in response to paclitaxel treatment.** FOXK2 and FOXO3a images from Figure 4a were quantified using ImageJ® analysis and plotted against signals for β-Tubulin.

**Supplementary Figure S5. FOXK2 is localised in the nucleus of FOXK2-overexpressing drug-sensitive and resistant cells.** MCF-7 and MCF-7Tax^R^ were transfected with the empty vector (pCMV5) and wild-type FOXK2 vector for 24 h, seeded on chamber slides and treated with 10 nM paclitaxel for 0 and 6 h. Cells were then fixed and immunostained for FOXK2 (green). Nuclei were counterstained with 4’-6-diamidino-2-phenyllindole (DAPI; blue). Images were acquired with Leica TCS SPS (x63 magnification). Images are representative of two independent experiments.

**Supplementary Figure S6. FOXK2 sensitizes MDA-MB-231 triple negative cells to paclitaxel. (a)** MDA-MB-231, MCF-7 and MCF-7Tax^R^ cells were exposed to 10 nM paclitaxel for 0, 6 and 24 h, harvested and subjected to western blot analysis, where FOXK2 and β-tubulin levels were determined. MDA-MB-231 cells were transfected with the empty (pCMV5) and wild-type FOXK2 vectors and efficiency of transfection was assessed by Western blotting and qRT-PCR. Following FOXK2 overexpression, MDA-MB-231 cells were seeded and treated with increasing concentrations of paclitaxel. Proliferation was measured by SRB **(b)** and clonogenic assays **(c)**. Bars represent average ± s.d. of three independent experiments. MDA-MB-231 cells overexpressing FOXK2 were treated with 10nM paclitaxel for 0, 6 and 24 h, after which they were harvested and collected for western blot analysis of FOXO3a expression **(d)** and qRT-PCT analysis of FOXO3a expression **(e)**. Bars represent average ± S.D. of three independent experiments. **(f)** MDA-MB-231 cells were transfected with NSC and FOXK2 siRNA sequences and efficiency of transfection was assessed by Western blotting. Following FOXK2 silencing, MDA-MB-231 were seeded and treated with increasing concentrations of paclitaxel. Proliferation was measured by SRB. Bars represent average ± S.D. of three independent experiments. Statistical significance was determined by Student’s t-test (* *p*≤0.05, ***p*≤0.01, ****p*≤0.001, significant).

**Supplementary Figure S7. FOXK2 depletion confers paclitaxel to MDA-MB-231 triple negative breast cancer cells.** MDA-MB-231 cells were transfected with NSC and FOXK2 siRNA and efficiency of transfection was assessed by Western blotting. Western blotting was performed to analyse for the expression of FOXK2 and β-tubulin after siRNA depletion. Following FOXK2 silencing, MDA-MB-231 were seeded and treated with increasing concentrations of paclitaxel. Proliferation was measured by SRB. Bars represent average ± S.D. of three independent experiments. Statistical significance was determined by Student’s t-test (* *p*≤0.05, ***p*≤0.01, significant).

**Supplementary Figure S8. FOXO3a is required for the anti-proliferative function of FOXK2 in MCF-7 breast cancer cells.**

MCF-7 cells were transiently transfected with either the control pCMV or FOXK2 and non-silencing control (NSC) siRNA or the FOXO3a siRNA smart pool. Twenty-four hours after transfection, aliquots of the transfected cells were split into 96 well plates and their proliferation analysed at the times indicated by SRB assays. Cell proliferation assays revealed that while MCF-7 cells transiently transfected with pFOXK2 proliferated slower than the control pCMV empty vector-transfected cells, there was no difference between pCMV and pFOXK2 transfected MCF-7 cells with FOXO3a silenced.

**Supplementary Figure S9.** **Correlation analysis between nuclear, cytoplasmic and total FOXK2 with total FOXO3a in all Invasive Ductal Carcinoma (IDC) patients.** Statistical analysis was performed using Chi-square Test: **P*≤0.05 = significant.

**Supplementary Figure S10.** **Correlation analysis between nuclear FOXK2 and total FOXO3a in Invasive Ductal Carcinoma (IDC) patients that received chemotehrapy.** Statistical analysis was performed using Chi-square Test: **P*≤0.05 = significant.

**Supplementary Figure S11.** **Correlation analysis between nuclear FOXK2 and the clinicopathological parameters of all patients.**

Correlation between nuclear FOXK2 and the clinicopathological parameters including ER status, PR status, tumour stage, histological type and lymph-node involvement. Nuclear FOXK2 expression was significantly associated with ER status and tumour stage. Statistical analysis was performed using Chi-square Test: **P*≤0.05 = significant.

**Supplementary Figure S12. Kaplan-Meier analysis showing cytoplasmic FOXK2 expression was not significantly correlated with patients’ survival in all IDC cases and in IDC patients who received chemotherapy.** **P*≤0.05 = significant.

**Supplementary Figure S13. A working model for the regulation of FOXO3a by FOXK2 in response to drug treatment in drug sensitive and resistant cells**
