# Supplementary material for: Forkhead box K2 modulates epirubicin and paclitaxel sensitivity through FOXO3a in breast cancer
Source: Oncogenesis. 2015 Sep 7;4(9):e167–. doi: 10.1038/oncsis.2015.26 (PMC4767938; doi:10.1038/oncsis.2015.26)
Supplement: Supplementary Materials and Methods [file oncsis201526x2.docx]

**Supplementary Materials and Methods**

***Tissue Microarray***

One hundred and thirty-three cases of breast cancer diagnosed between the years 1992 to 2001 with clinical follow up data were retrieved from the records of the Department of Pathology, Queen Mary Hospital of Hong Kong, with approval by the Institutional Review Board of The University of Hong Kong with patient consents. Histological sections of all cases were reviewed by the pathologist, the representative paraffin tumour blocks chosen as donor block for each case and the selected areas marked for construction of tissue microarray (TMA) blocks. A total of 116 could be assessed and scored for FOXO3a and FOXK2 staining. The expression pattern and subcellular localization were correlated with various clinicopathological data including ER, PR status, age, histological grade, histological type, clinical stage, lymph node metastasis as well as survival time.

***Immunohistochemistry***

The TMA sections were deparaffinised and rehydrated by incubation with xylene and decreasing concentrations of ethanol. Citrate buffer (0.01M, pH 6.0) was used for antigen retrieval. The slides were immersed into 3% H_2_O_2_/methanol for 10 min at room temperature to quench endogenous peroxidase. After rinsing in 0.05% Tween in PBS (PBST) twice, a previously described FOXO3a antibody {Chen, 2010 #34} diluted at 1:1400 or a FOXK2 specific antibody at 1:50 (Bethyl Labs) was added to each section and incubated at 4°C overnight. The slides were then washed in PBST and incubated with DAKO EnVision+System-HRP-labelled Polymer Anti-Rabbit at room temperature in dark for 30 min. After washing, Chromogen DAB/substrate reagent was added onto the slides and the slides incubated for a further 6 minutes. Finally, the slides were dehydrated and mounted. Aperio ScanScope ® system (Aperio technology, USA) was used to visualize and assess for FOXO3a and FOXK2 expression.

***Scoring of IHC staining***

The staining of FOXO3a and FOXK2 were assessed by light microscope visualized at high power (X170). The staining intensity and percentage of staining in the cytoplasm and the nucleus were scored in a semi-quantitative fashion. The intensity of staining was scored as follows: 1 =  weak, 2 =  moderate, 3 =  strong. The percentage of cells positively stained was scored as follows: 1≤25%, 2≤50%, 3≤75%, 4>75%. For each case, a final score from the nucleus and the cytoplasm was obtained by multiplying the score of intensity with the score of percentage, 12 being the maximum final score. To avoid subjectivity in evaluation, scoring was done by two independent individuals.

**Chromatin immunoprecipitation (ChIP)**

Cells were cross-linked by the addition of 1% formaldehyde and incubated for 10 min at RT, after which the reaction was stopped with incubation with 2.5M glycine for 5 min (final concentration: 0.125 M). Cells were then washed in PBS and 1 mL of PBS containing protease inhibitors was added. Cells were scraped, transferred to an eppendorf tube and centrifuged at 3000 rpm for 3 min. Pellets were resuspended in 1mL of lysis buffer 1 containing protease inhibitors (50mM HEPES pH 7.5, 140mM NaCl, 1mM EDTA, 10% glycerol, 0.5% Igepal CA-630, 0.25% Triton-X), rotated at 4°C for 10 min and centrifuged at 2000 g for 5 min at 4°C. Supernatant was poured off and 1 mL lysis buffer 2 containing protease inhibitors (200mM NaCl, 1mM EDTA, 0.5mM EGTA, 10mM Tris-HCl pH 8.0) was added to the pellets, which were rotated for 5 min and then centriguged again. Pellets were then resuspended in 300 µL of lysis buffer 3 containing protease inhibitors (100mM NaCl, 1mM EDTA, 0.5mM EGTA, 10mM Tris-HCl pH 8.0, 0.1% Na-deoxycholate, 0.5% N-lauroylsarcosin) and sonicated using a Bioruptor™ Next Gen sonicator (Diagenode) for 5 pulses of 30 s, after which Triton-X was added to the sonicated lysates to a final concentration of 1 x. Following centrifugation at full speed for 10 min, supernatants were collected for INPUT (15 µL) and pull-down (100 µL per pull down). For the immunoprecipitation, 4 µg of either IgG (P0447, Dako; Cambridgeshire, UK) and FOXK2 (ab5298; Abcam; Cambridge, Massachussetts, USA) Santa Cruz Biotechnology; Heidelberg, Germany) antibodies were added to the precleared samples, rotated overnight at 4°C and washed 6x with RIPA buffer (50mM HEPES pH 7.6, 1mM EDTA, 0.7% Na-deoxycholate, 1% Igepal CA-630, 0.5M LiCl) and 2x with TE buffer. The antibody-containing beads were resuspended in 100 µL of elution buffer (1% SDS, 0.1M NaHCO_3_) and incubated at 65°C overnight. Decrosslinking was performed by the addition of 200 µL of TE buffer containing 8 µL of RNAse (1mg/ mL) and incubation for 1 h at 37°C. For digestion, 4 µL of proteinase K was then added followed by a 2-h incubation at 55°C and phenol-chloroform DNA extraction (Sigma; St. Louis, MO, US). For DNA precipitation, 30 µL of 5M NaCl, 1 µL of glycoblue (Ambion) and 900 µL of 100% ethanol (3x the volume) were added to the tubes, which were kept at -80°C for at least 1 night. On the following day, the pellets were washed with 100% ethanol and then quantified using the picogreen reagent (Life Technologies). Fluorescence was measured in a PHERAstar^Plus^ microplate reader (BMG Labtech, Ortenberg, Germany). Then, we used 2.5 µL DNA from each sample, 0.5 µL of mix of primers (50 nM final concentration), 5 µL SYBR green master mix (Applied Biosystems) and 2 µL DEPC-treated water per well. The reaction was run in 7900 HT Fast Real-time PCR System(Applied Biosystems) and the cycling program was 95°C for 10 min followed by 40 cycles of 95°C for 15 s, 60°C for 30 s and 95°C for 30 s, followed by a dissociation step. The pair of primers used for ChIP was: FOXO3a-F, 5’-ACCAACATCTTCGCCGTTC-3’ and FOXO3a-R, 5’-GGTGTCCGGTTCCCTGTTAG-3’, Each sample was assayed in triplicates, and the results were normalized to the IgG antibody.
