# Supplementary material for: Forkhead box K2 modulates epirubicin and paclitaxel sensitivity through FOXO3a in breast cancer
Source: Oncogenesis. 2015 Sep 7;4(9):e167–. doi: 10.1038/oncsis.2015.26 (PMC4767938; doi:10.1038/oncsis.2015.26)
Supplement: Supplementary Figure 1 [file oncsis201526x3.ppt]

## Slide 1
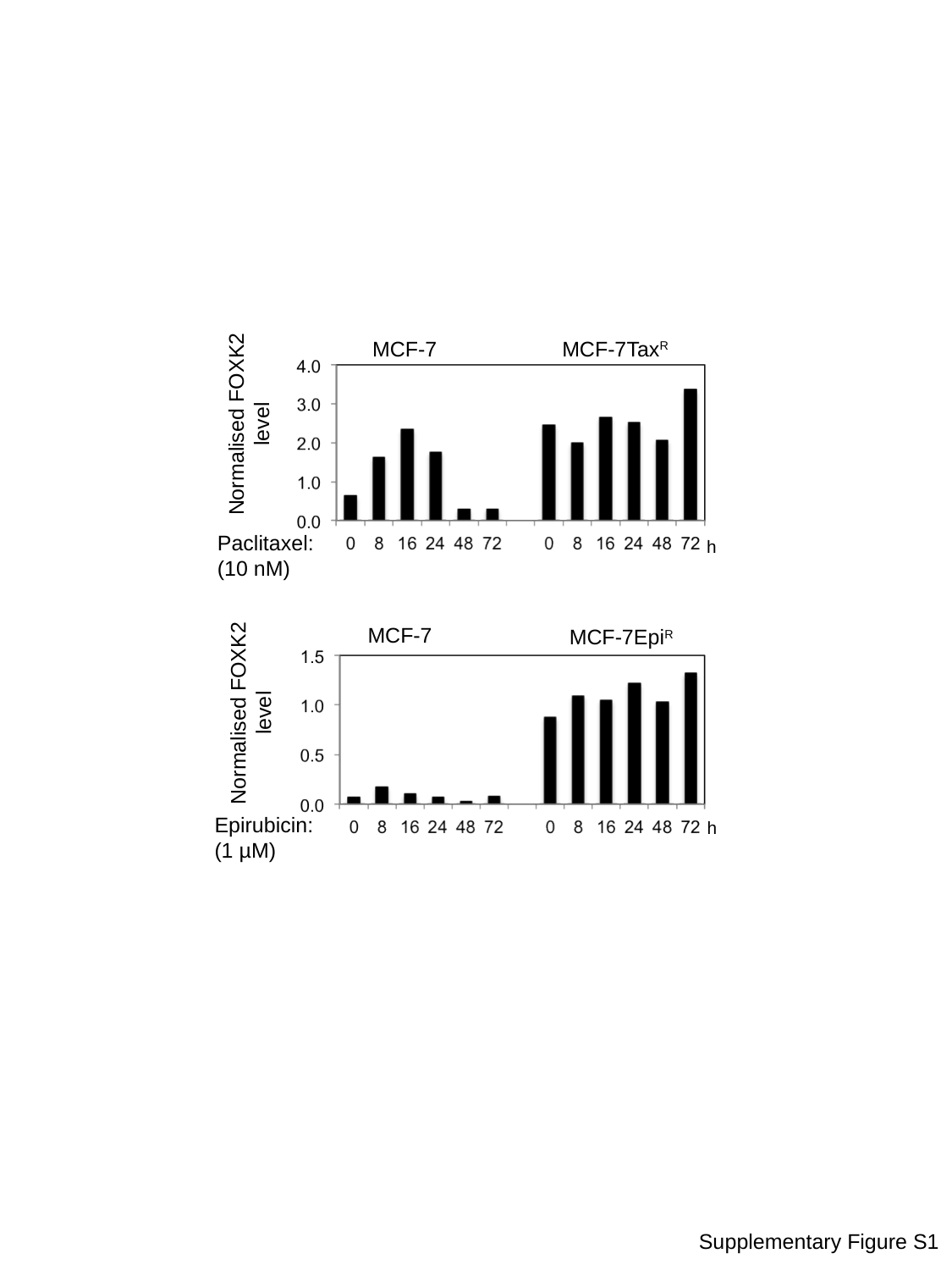

MCF-7TaxR
MCF-7
Normalised FOXK2
level
Paclitaxel:
(10 nM)
h
MCF-7
MCF-7EpiR
Normalised FOXK2
level
Epirubicin:
(1 µM)
h
Supplementary Figure S1
