# Supplementary material for: Forkhead box K2 modulates epirubicin and paclitaxel sensitivity through FOXO3a in breast cancer
Source: Oncogenesis. 2015 Sep 7;4(9):e167–. doi: 10.1038/oncsis.2015.26 (PMC4767938; doi:10.1038/oncsis.2015.26)
Supplement: Supplementary Figure 2 [file oncsis201526x4.ppt]

## Slide 1
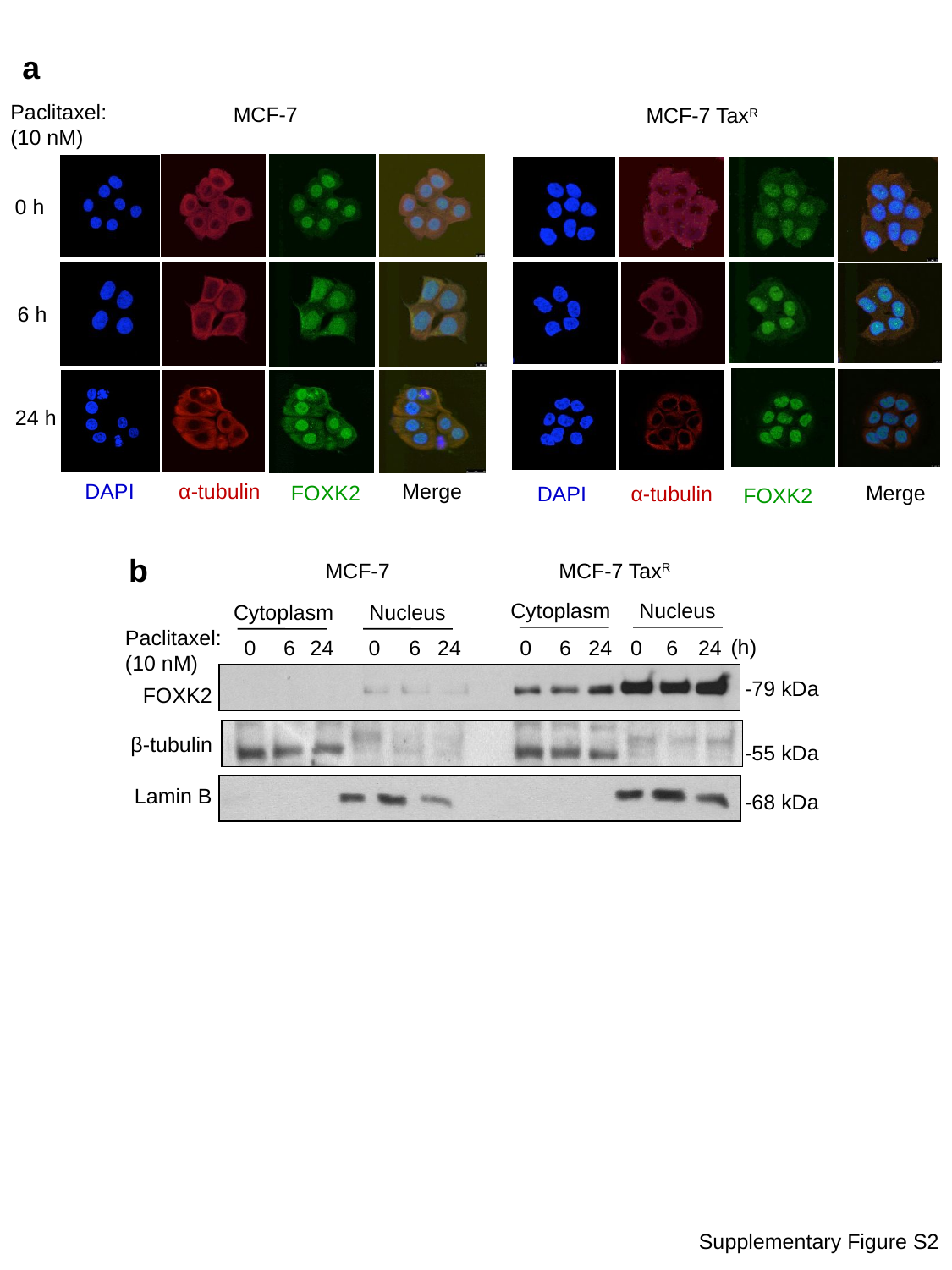

a
Paclitaxel:
(10 nM)
MCF-7
MCF-7 TaxR
0 h
6 h
24 h
Merge
DAPI
α-tubulin
FOXK2
Merge
DAPI
α-tubulin
FOXK2
b
MCF-7
MCF-7 TaxR
Cytoplasm
Nucleus
Cytoplasm
Nucleus
Paclitaxel:
(10 nM)
(h)
0
6
24
0
6
24
0
6
24
0
6
24
-79 kDa
FOXK2
β-tubulin
-55 kDa
Lamin B
-68 kDa
Supplementary Figure S2
