# Supplementary material for: Forkhead box K2 modulates epirubicin and paclitaxel sensitivity through FOXO3a in breast cancer
Source: Oncogenesis. 2015 Sep 7;4(9):e167–. doi: 10.1038/oncsis.2015.26 (PMC4767938; doi:10.1038/oncsis.2015.26)
Supplement: Supplementary Figure 3 [file oncsis201526x5.ppt]

## Slide 1
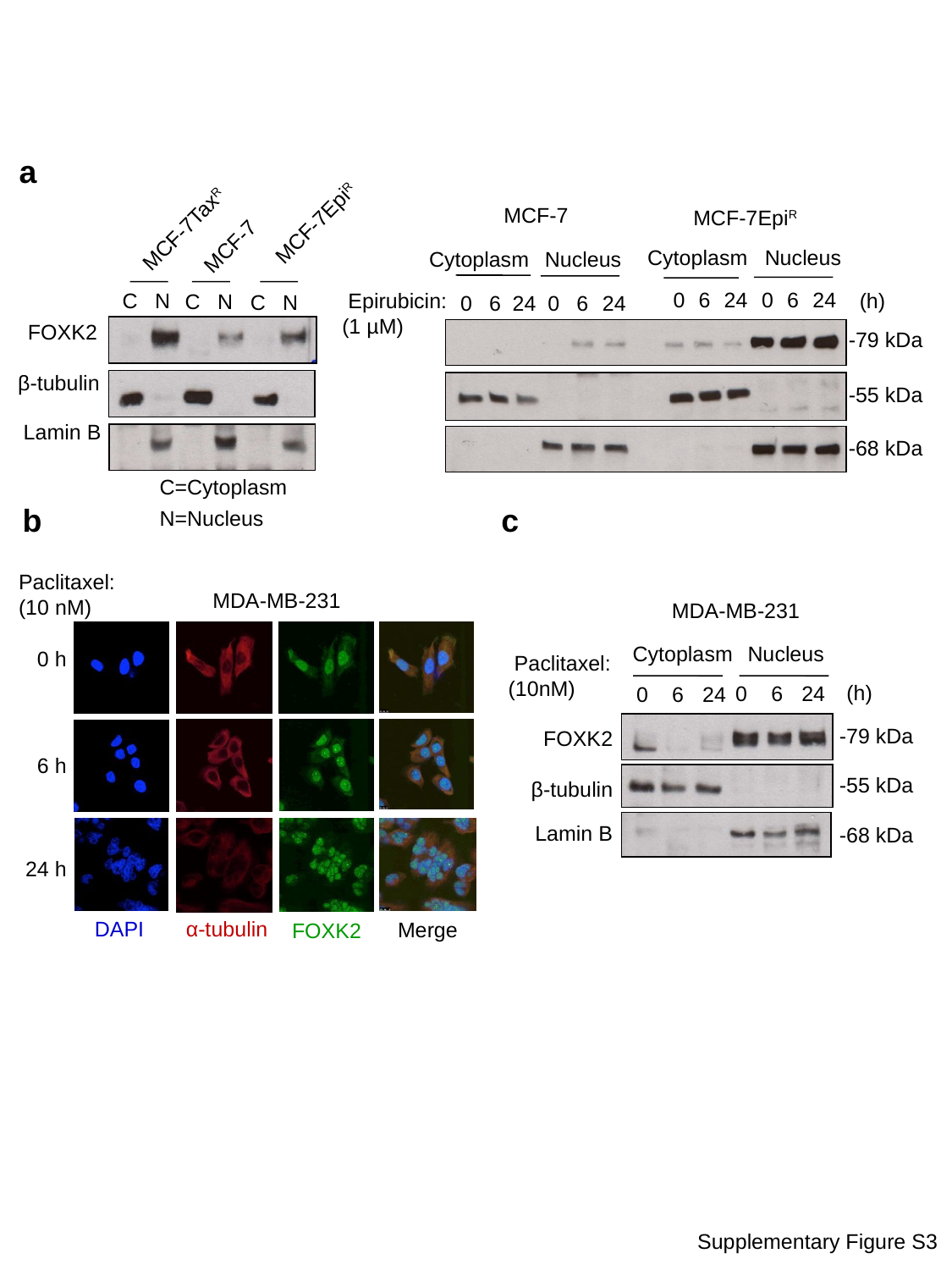

a
MCF-7
MCF-7EpiR
MCF-7EpiR
MCF-7TaxR
MCF-7
Cytoplasm
Nucleus
Cytoplasm
Nucleus
0
6
24
0
6
24
C
N
 Epirubicin:
(1 µM)
(h)
C
N
C
N
0
6
24
0
6
24
FOXK2
-79 kDa
β-tubulin
-55 kDa
Lamin B
-68 kDa
C=Cytoplasm
b
c
N=Nucleus
Paclitaxel:
(10 nM)
MDA-MB-231
MDA-MB-231
Cytoplasm
Nucleus
0 h
 Paclitaxel:
(10nM)
(h)
0
6
24
0
6
24
-79 kDa
FOXK2
6 h
-55 kDa
β-tubulin
Lamin B
-68 kDa
24 h
DAPI
α-tubulin
Merge
FOXK2
Supplementary Figure S3
