# Supplementary material for: Forkhead box K2 modulates epirubicin and paclitaxel sensitivity through FOXO3a in breast cancer
Source: Oncogenesis. 2015 Sep 7;4(9):e167–. doi: 10.1038/oncsis.2015.26 (PMC4767938; doi:10.1038/oncsis.2015.26)
Supplement: Supplementary Figure 4 [file oncsis201526x6.ppt]

## Slide 1
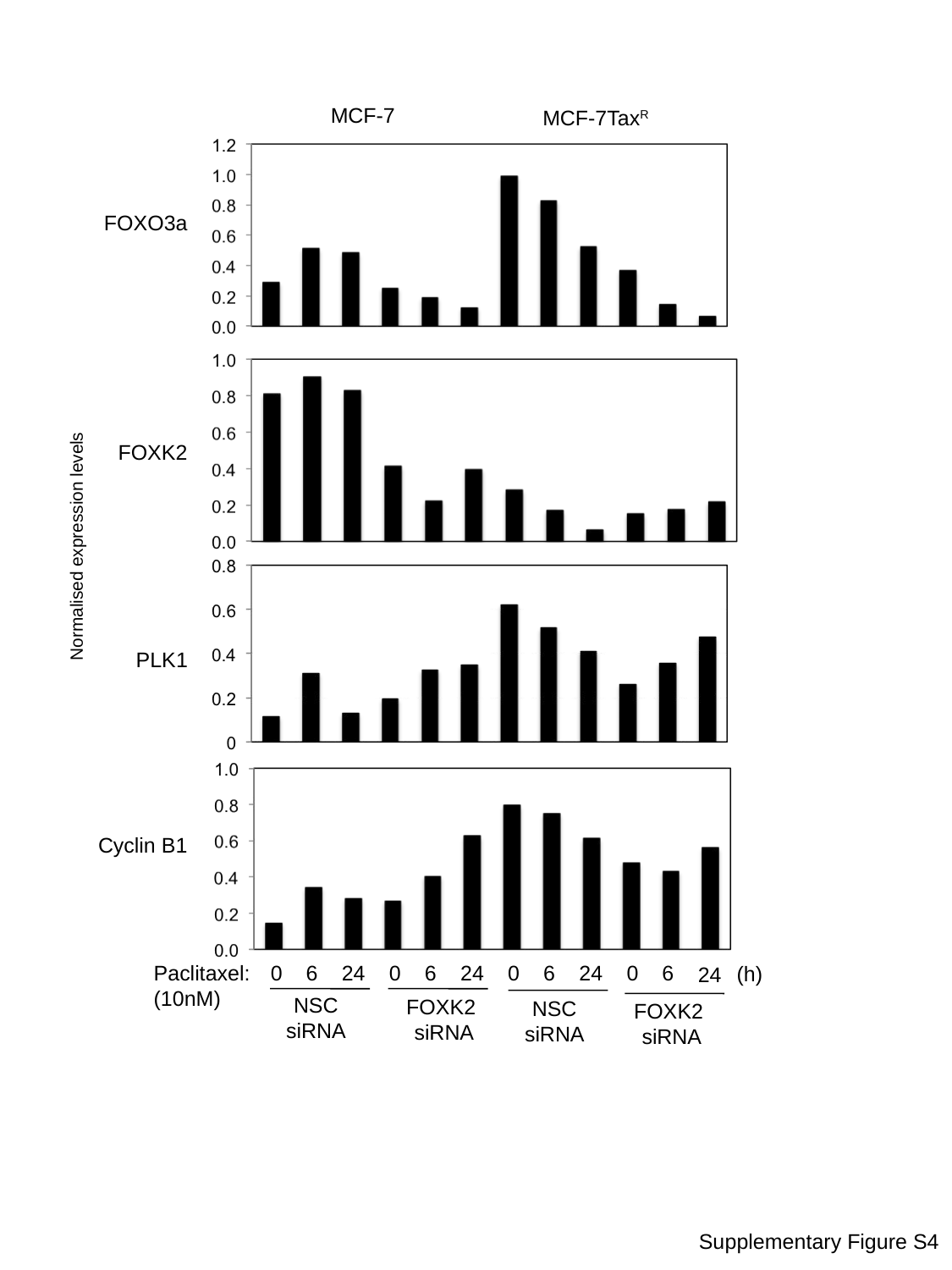

MCF-7
MCF-7TaxR
FOXO3a
FOXK2
Normalised expression levels
PLK1
Cyclin B1
Paclitaxel:
(10nM)
0
6
24
0
6
24
0
6
24
0
6
(h)
24
NSC
siRNA
FOXK2
siRNA
NSC
siRNA
FOXK2
siRNA
Supplementary Figure S4
