# Supplementary material for: Forkhead box K2 modulates epirubicin and paclitaxel sensitivity through FOXO3a in breast cancer
Source: Oncogenesis. 2015 Sep 7;4(9):e167–. doi: 10.1038/oncsis.2015.26 (PMC4767938; doi:10.1038/oncsis.2015.26)
Supplement: Supplementary Figure 5 [file oncsis201526x7.ppt]

## Slide 1
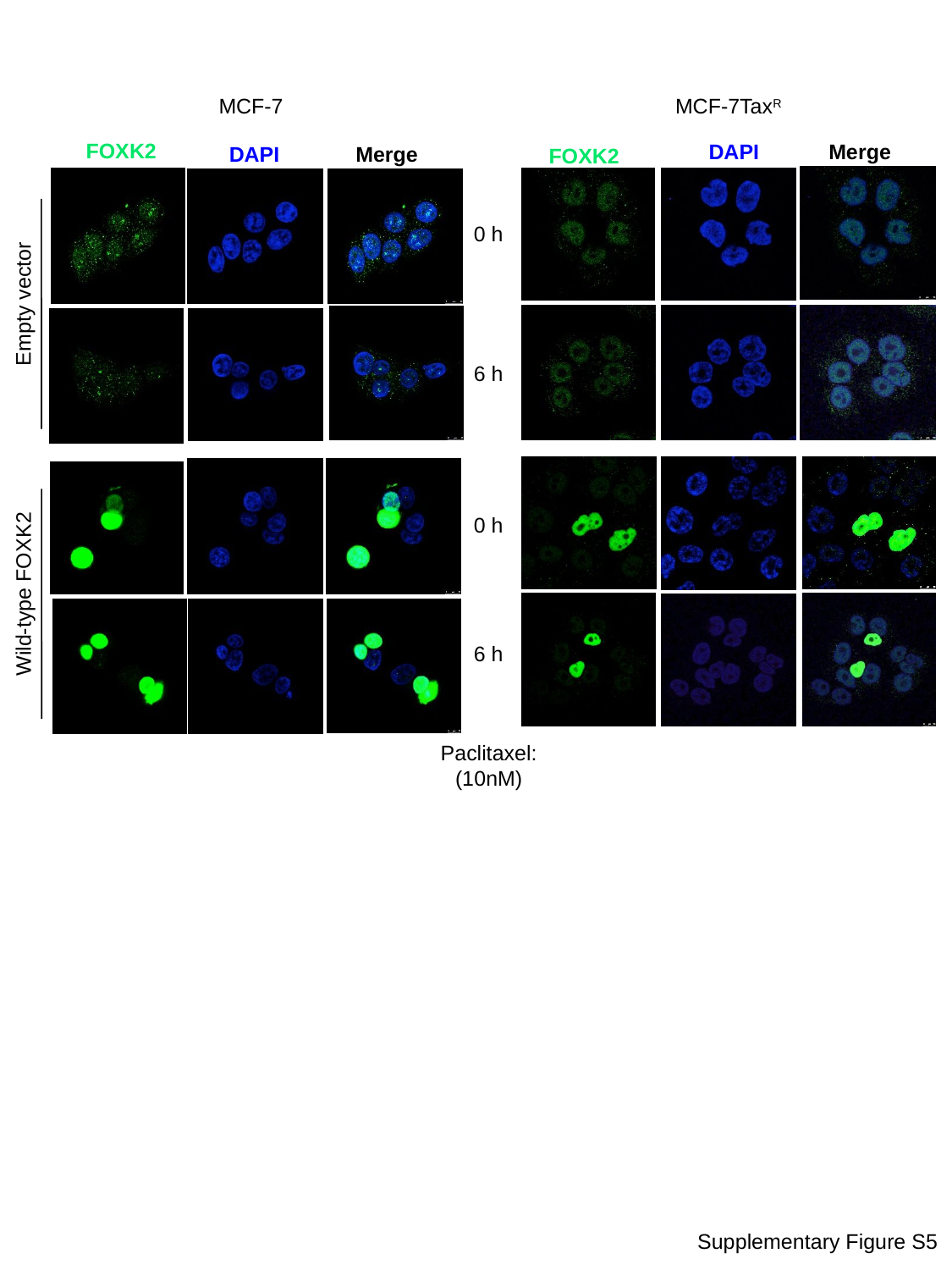

MCF-7
MCF-7TaxR
FOXK2
DAPI
Merge
DAPI
Merge
FOXK2
0 h
Empty vector
6 h
0 h
Wild-type FOXK2
6 h
Paclitaxel:
 (10nM)
Supplementary Figure S5
