# Supplementary material for: Forkhead box K2 modulates epirubicin and paclitaxel sensitivity through FOXO3a in breast cancer
Source: Oncogenesis. 2015 Sep 7;4(9):e167–. doi: 10.1038/oncsis.2015.26 (PMC4767938; doi:10.1038/oncsis.2015.26)
Supplement: Supplementary Figure 6 [file oncsis201526x8.ppt]

## Slide 1
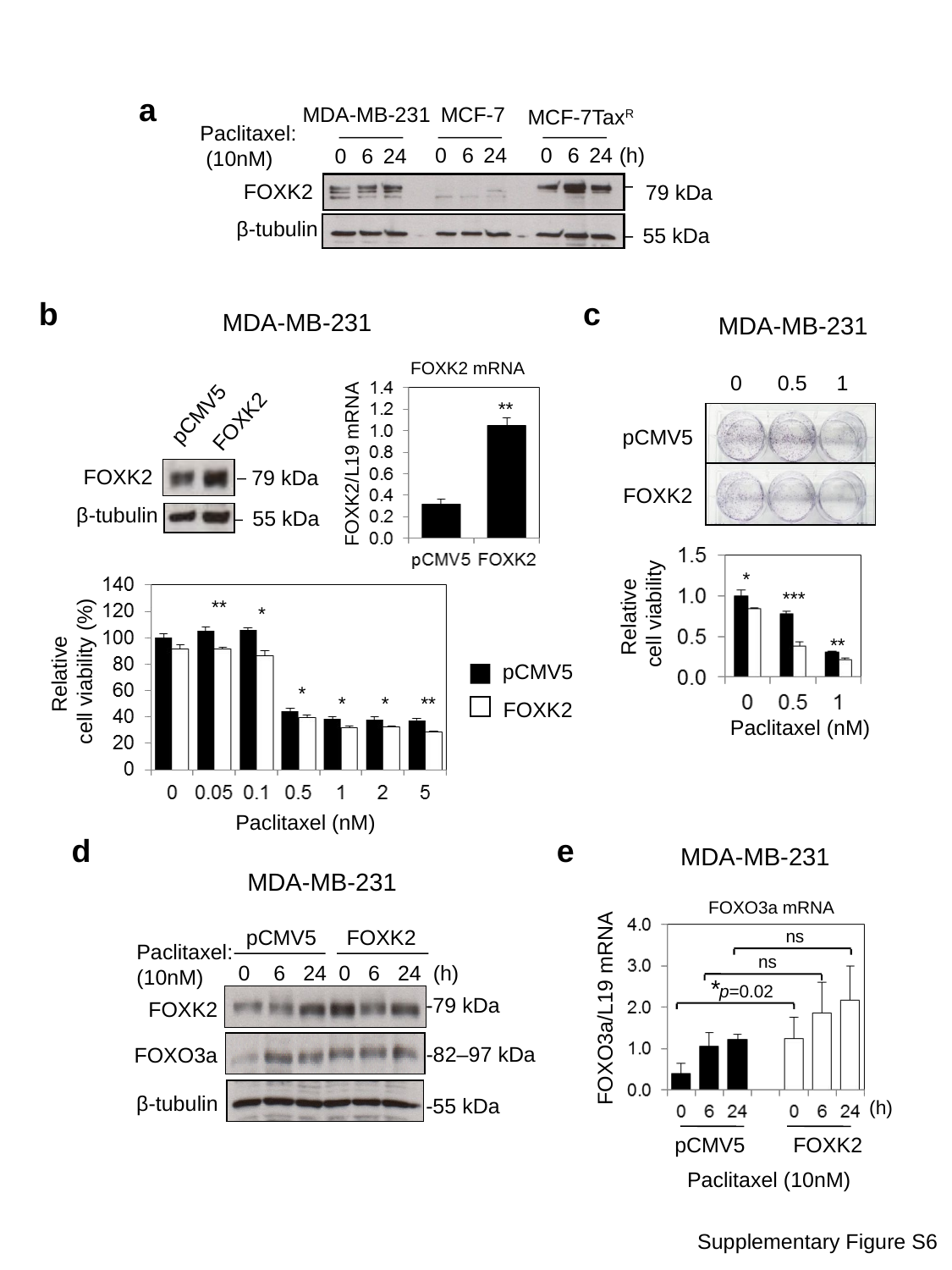

a
MDA-MB-231
MCF-7
MCF-7TaxR
Paclitaxel:
 (10nM)
(h)
0
6
24
0
6
24
0
6
24
FOXK2
79 kDa
β-tubulin
55 kDa
b
c
MDA-MB-231
MDA-MB-231
FOXK2 mRNA
 0 0.5 1
**
pCMV5
FOXK2
pCMV5
FOXK2/L19 mRNA
FOXK2
79 kDa
FOXK2
β-tubulin
55 kDa
*
***
Relative
cell viability
**
*
**
Relative
cell viability (%)
pCMV5
FOXK2
*
*
*
**
 Paclitaxel (nM)
 Paclitaxel (nM)
d
e
MDA-MB-231
MDA-MB-231
FOXO3a mRNA
pCMV5
FOXK2
ns
Paclitaxel:
(10nM)
ns
0 6 24 0 6 24 (h)
*p=0.02
-79 kDa
FOXO3a/L19 mRNA
FOXK2
-82–97 kDa
FOXO3a
β-tubulin
-55 kDa
(h)
pCMV5
FOXK2
Paclitaxel (10nM)
Supplementary Figure S6
