# Supplementary material for: Forkhead box K2 modulates epirubicin and paclitaxel sensitivity through FOXO3a in breast cancer
Source: Oncogenesis. 2015 Sep 7;4(9):e167–. doi: 10.1038/oncsis.2015.26 (PMC4767938; doi:10.1038/oncsis.2015.26)
Supplement: Supplementary Figure 7 [file oncsis201526x9.ppt]

## Slide 1
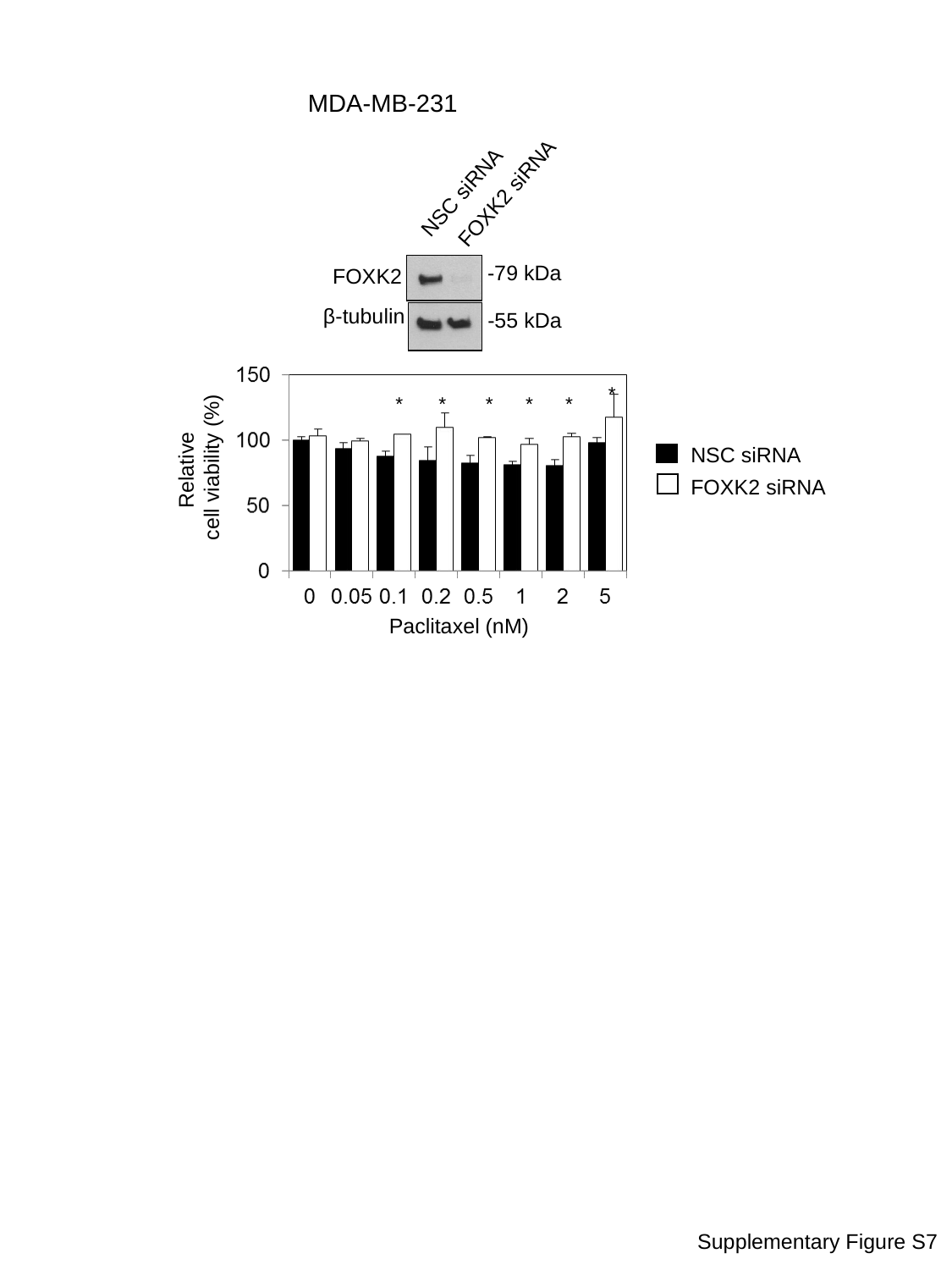

MDA-MB-231
NSC siRNA
FOXK2 siRNA
-79 kDa
FOXK2
β-tubulin
-55 kDa
*
*
*
*
*
*
NSC siRNA
Relative
cell viability (%)
FOXK2 siRNA
 Paclitaxel (nM)
Supplementary Figure S7
