# Supplementary material for: Forkhead box K2 modulates epirubicin and paclitaxel sensitivity through FOXO3a in breast cancer
Source: Oncogenesis. 2015 Sep 7;4(9):e167–. doi: 10.1038/oncsis.2015.26 (PMC4767938; doi:10.1038/oncsis.2015.26)
Supplement: Supplementary Figure 8 [file oncsis201526x10.ppt]

## Slide 1
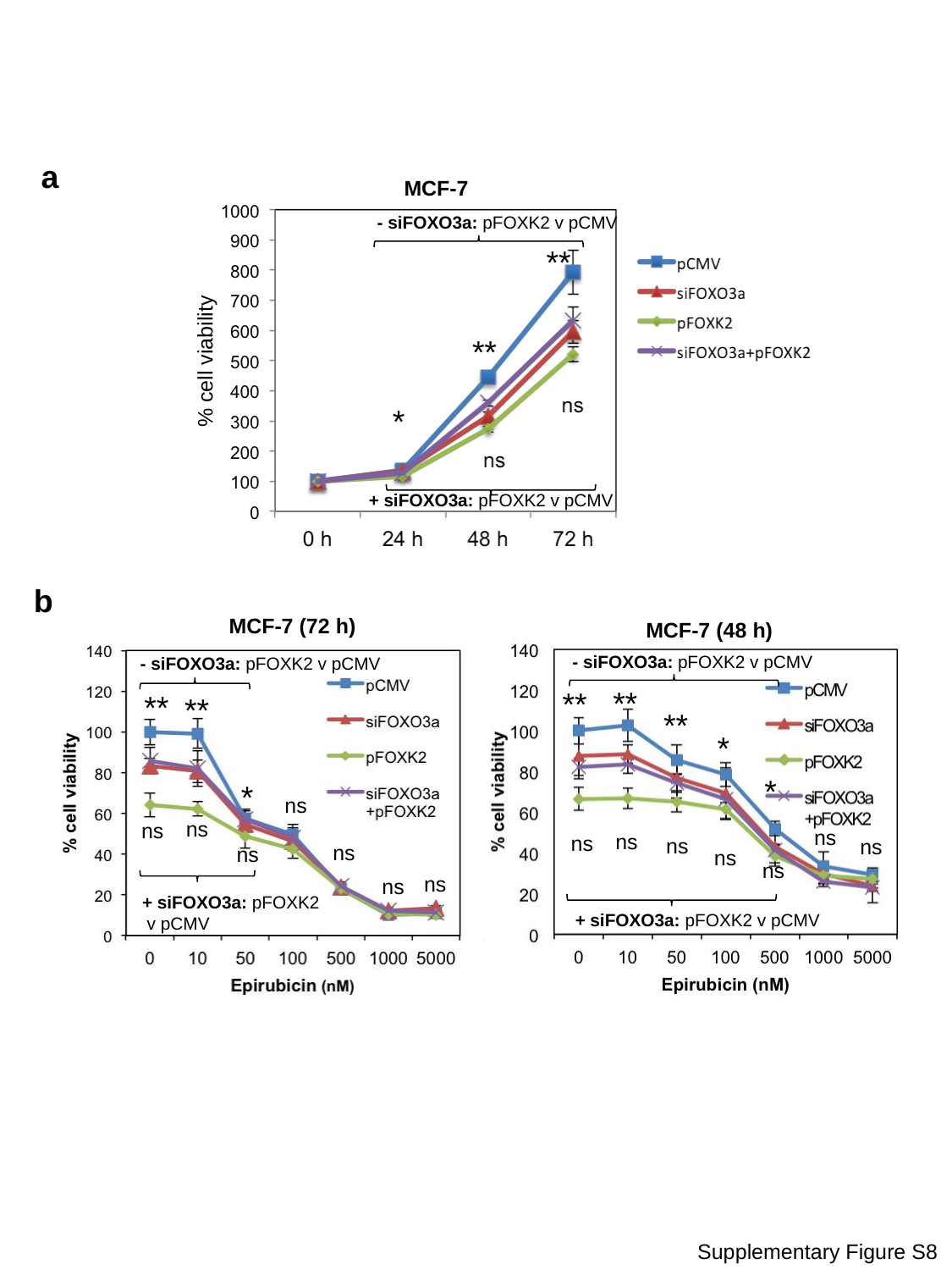

a
MCF-7
- siFOXO3a: pFOXK2 v pCMV
**
**
% cell viability
*
+ siFOXO3a: pFOXK2 v pCMV
b
MCF-7 (72 h)
MCF-7 (48 h)
- siFOXO3a: pFOXK2 v pCMV
- siFOXO3a: pFOXK2 v pCMV
**
**
**
**
*
*
*
*
*
*
**
*
NSC siRNA
Relative
cell viability (%)
*
*
FOXK2 siRNA
ns
ns
ns
ns
ns
ns
ns
ns
ns
ns
ns
ns
ns
ns
+ siFOXO3a: pFOXK2
 v pCMV
+ siFOXO3a: pFOXK2 v pCMV
 Paclitaxel (nM)
Supplementary Figure S8
