# Supplementary material for: Forkhead box K2 modulates epirubicin and paclitaxel sensitivity through FOXO3a in breast cancer
Source: Oncogenesis. 2015 Sep 7;4(9):e167–. doi: 10.1038/oncsis.2015.26 (PMC4767938; doi:10.1038/oncsis.2015.26)
Supplement: Supplementary Figure 9 [file oncsis201526x11.ppt]

## Slide 1
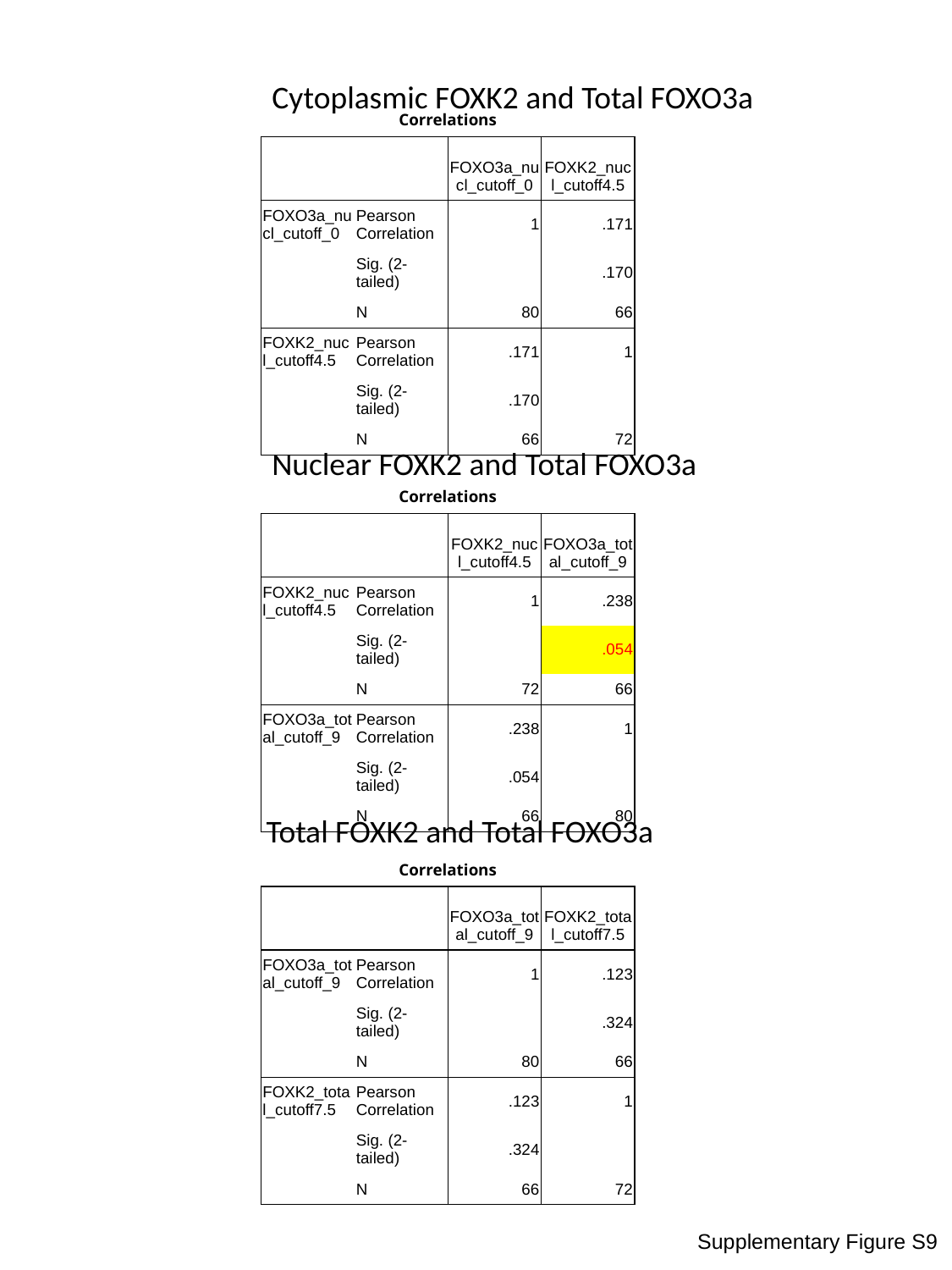

Cytoplasmic FOXK2 and Total FOXO3a
| Correlations | | | |
| --- | --- | --- | --- |
| | | FOXO3a\_nucl\_cutoff\_0 | FOXK2\_nucl\_cutoff4.5 |
| FOXO3a\_nucl\_cutoff\_0 | Pearson Correlation | 1 | .171 |
| | Sig. (2-tailed) | | .170 |
| | N | 80 | 66 |
| FOXK2\_nucl\_cutoff4.5 | Pearson Correlation | .171 | 1 |
| | Sig. (2-tailed) | .170 | |
| | N | 66 | 72 |
Nuclear FOXK2 and Total FOXO3a
| Correlations | | | |
| --- | --- | --- | --- |
| | | FOXK2\_nucl\_cutoff4.5 | FOXO3a\_total\_cutoff\_9 |
| FOXK2\_nucl\_cutoff4.5 | Pearson Correlation | 1 | .238 |
| | Sig. (2-tailed) | | .054 |
| | N | 72 | 66 |
| FOXO3a\_total\_cutoff\_9 | Pearson Correlation | .238 | 1 |
| | Sig. (2-tailed) | .054 | |
| | N | 66 | 80 |
Total FOXK2 and Total FOXO3a
| Correlations | | | |
| --- | --- | --- | --- |
| | | FOXO3a\_total\_cutoff\_9 | FOXK2\_total\_cutoff7.5 |
| FOXO3a\_total\_cutoff\_9 | Pearson Correlation | 1 | .123 |
| | Sig. (2-tailed) | | .324 |
| | N | 80 | 66 |
| FOXK2\_total\_cutoff7.5 | Pearson Correlation | .123 | 1 |
| | Sig. (2-tailed) | .324 | |
| | N | 66 | 72 |
Supplementary Figure S9
