# Supplementary figures and images for: Forkhead box K2 modulates epirubicin and paclitaxel sensitivity through FOXO3a in breast cancer
Source: Oncogenesis. 2015 Sep 7;4(9):e167–. doi: 10.1038/oncsis.2015.26 (PMC4767938; doi:10.1038/oncsis.2015.26)

## Slide 1
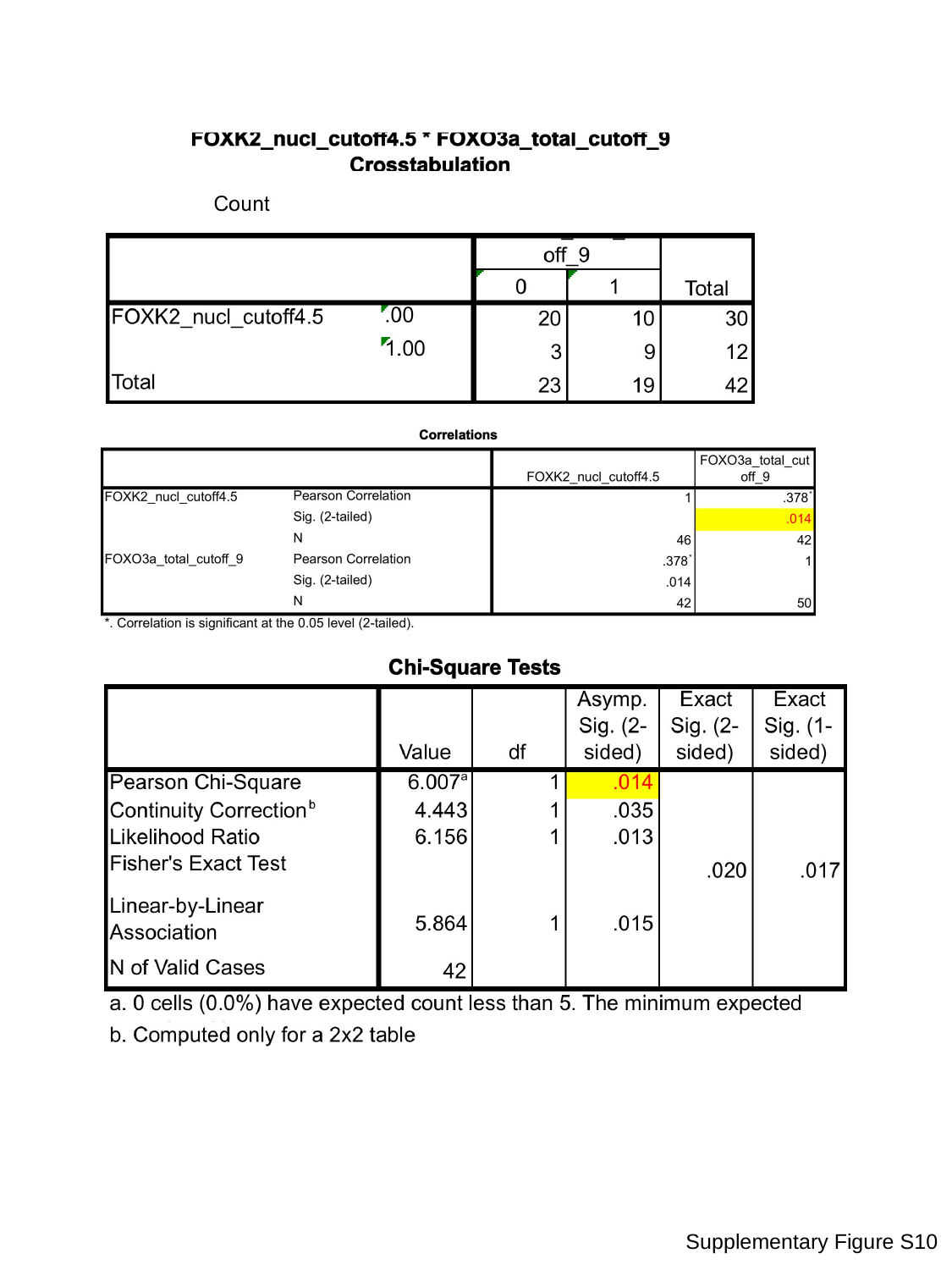

Supplementary Figure S10

Supplement: Supplementary Figure 10 [file oncsis201526x12.ppt]
