# Supplementary material for: Forkhead box K2 modulates epirubicin and paclitaxel sensitivity through FOXO3a in breast cancer
Source: Oncogenesis. 2015 Sep 7;4(9):e167–. doi: 10.1038/oncsis.2015.26 (PMC4767938; doi:10.1038/oncsis.2015.26)
Supplement: Supplementary Figure 11 [file oncsis201526x13.ppt]

## Slide 1
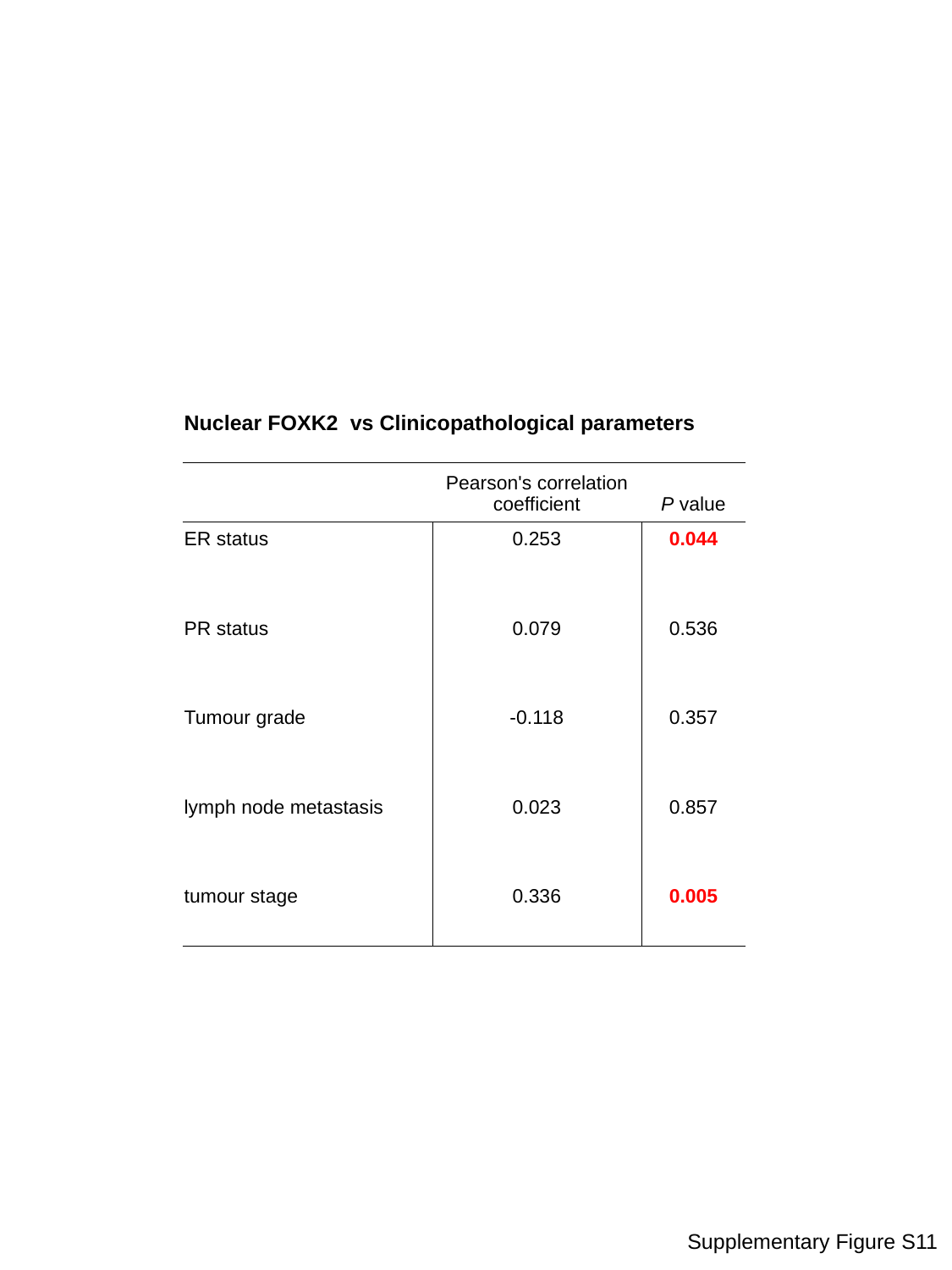

| Nuclear FOXK2 vs Clinicopathological parameters | | |
| --- | --- | --- |
| | Pearson's correlation coefficient | P value |
| ER status | 0.253 | 0.044 |
| | | |
| PR status | 0.079 | 0.536 |
| | | |
| Tumour grade | -0.118 | 0.357 |
| | | |
| lymph node metastasis | 0.023 | 0.857 |
| | | |
| tumour stage | 0.336 | 0.005 |
| | | |
Supplementary Figure S11
