# Supplementary material for: Forkhead box K2 modulates epirubicin and paclitaxel sensitivity through FOXO3a in breast cancer
Source: Oncogenesis. 2015 Sep 7;4(9):e167–. doi: 10.1038/oncsis.2015.26 (PMC4767938; doi:10.1038/oncsis.2015.26)
Supplement: Supplementary Figure 12 [file oncsis201526x14.ppt]

## Slide 1
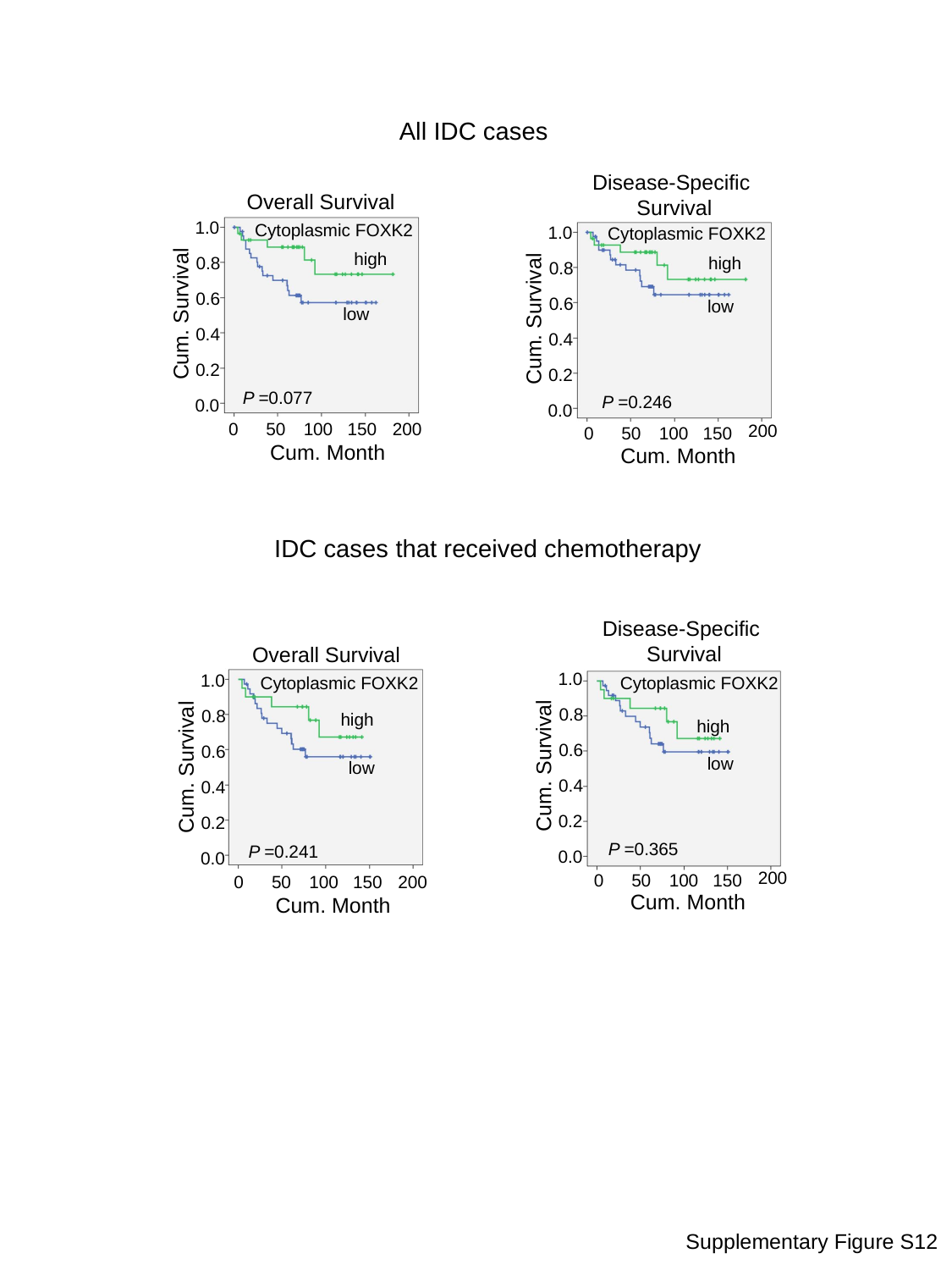

All IDC cases
Disease-Specific
Survival
1.0
Cytoplasmic FOXK2
Total FOXK2
high
0.8
low
0.6
low
Cum. Survival
0.4
high
0.2
P =0.246
P =0.58
0.0
200
0
50
100
150
Cum. Month
Overall Survival
1.0
Cytoplasmic FOXK2
high
0.8
0.6
Cum. Survival
low
0.4
0.2
P =0.077
0.0
0
50
100
150
200
Cum. Month
IDC cases that received chemotherapy
Disease-Specific
Survival
1.0
Cytoplasmic FOXK2
0.8
high
0.6
low
Cum. Survival
0.4
0.2
P =0.365
0.0
200
0
50
100
150
Cum. Month
Overall Survival
1.0
Cytoplasmic FOXK2
0.8
high
0.6
Cum. Survival
low
0.4
0.2
P =0.241
0.0
0
50
100
150
200
Cum. Month
Supplementary Figure S12
