# Supplementary material for: Forkhead box K2 modulates epirubicin and paclitaxel sensitivity through FOXO3a in breast cancer
Source: Oncogenesis. 2015 Sep 7;4(9):e167–. doi: 10.1038/oncsis.2015.26 (PMC4767938; doi:10.1038/oncsis.2015.26)
Supplement: Supplementary Figure 13 [file oncsis201526x15.ppt]

## Slide 1
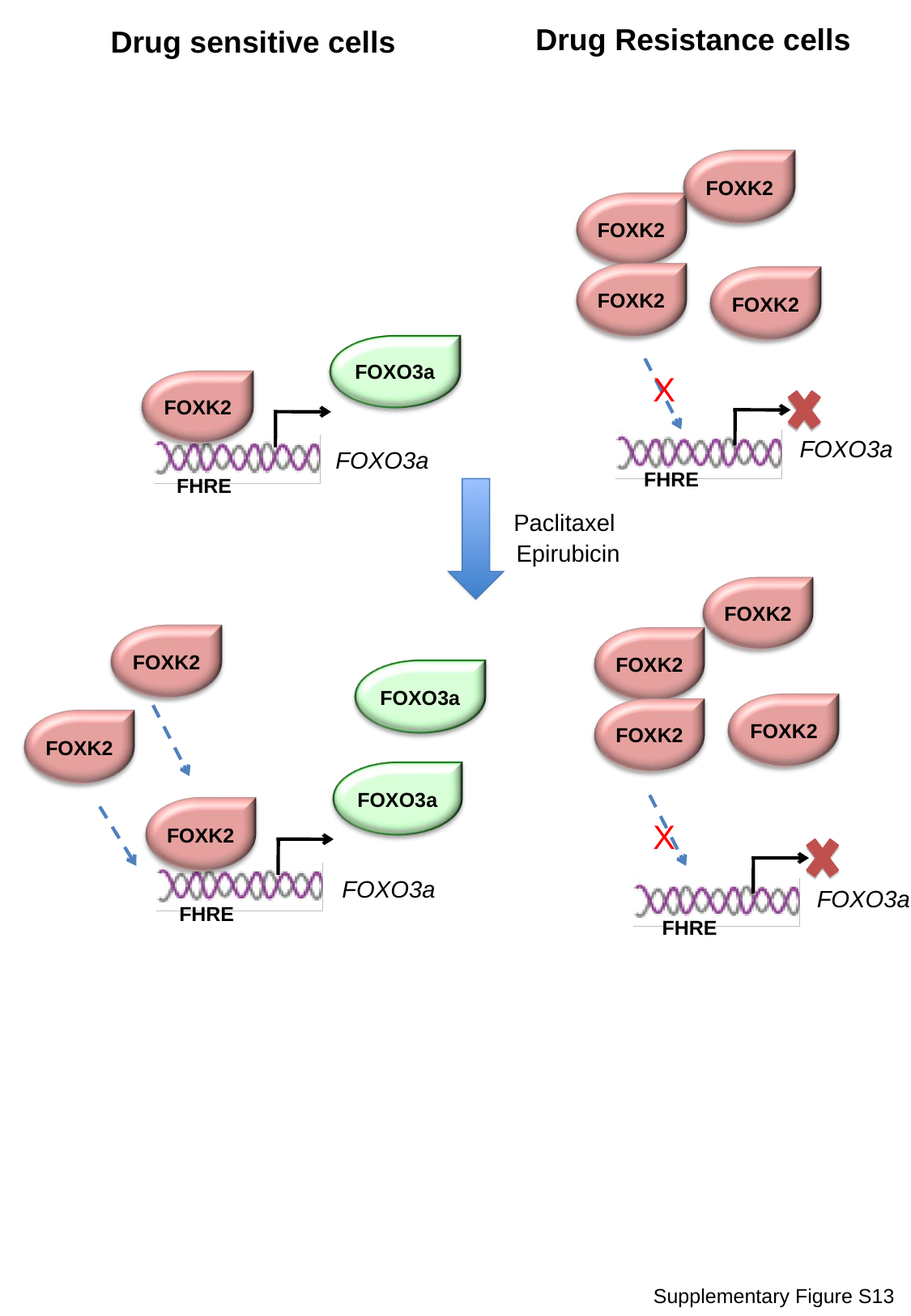

Drug Resistance cells
Drug sensitive cells
FOXK2
FOXK2
FOXK2
FOXK2
FOXO3a
X
FOXK2
FOXO3a
FOXO3a
FHRE
FHRE
Paclitaxel
Epirubicin
FOXK2
FOXK2
FOXK2
FOXO3a
FOXK2
FOXK2
FOXK2
FOXO3a
FOXK2
X
FOXO3a
FOXO3a
FHRE
FHRE
Supplementary Figure S13
